# Scientific Production Dynamics in mHealth for Diabetes: Scientometric Analysis

**DOI:** 10.2196/52196

**Published:** 2024-08-22

**Authors:** Pedro Fernando Castillo-Valdez, Marisela Rodriguez-Salvador, Yuh-Shan Ho

**Affiliations:** 1 Tecnologico de Monterrey Monterrey Mexico; 2 CT HO Trend Taipei Taiwan

**Keywords:** competitive technology intelligence, diabetes, digital health care, mobile health, mHealth, scientometrics, mobile phone

## Abstract

**Background:**

The widespread use of mobile technologies in health care (mobile health; mHealth) has facilitated disease management, especially for chronic illnesses such as diabetes. mHealth for diabetes is an attractive alternative to reduce costs and overcome geographical and temporal barriers to improve patients’ conditions.

**Objective:**

This study aims to reveal the dynamics of scientific publications on mHealth for diabetes to gain insights into who are the most prominent authors, countries, institutions, and journals and what are the most cited documents and current hot spots.

**Methods:**

A scientometric analysis based on a competitive technology intelligence methodology was conducted. An innovative 8-step methodology supported by experts was executed considering scientific documents published between 1998 and 2021 in the Science Citation Index Expanded database. Publication language, publication output characteristics, journals, countries and institutions, authors, and most cited and most impactful articles were identified.

**Results:**

The insights obtained show that a total of 1574 scientific articles were published by 7922 authors from 90 countries, with an average of 15 (SD 38) citations and 6.5 (SD 4.4) authors per article. These documents were published in 491 journals and 92 Web of Science categories. The most productive country was the United States, followed by the United Kingdom, China, Australia, and South Korea, and the top 3 most productive institutions came from the United States, whereas the top 3 most cited articles were published in 2016, 2009, and 2017 and the top 3 most impactful articles were published in 2016 and 2017.

**Conclusions:**

This approach provides a comprehensive knowledge panorama of research productivity in mHealth for diabetes, identifying new insights and opportunities for research and development and innovation, including collaboration with other entities, new areas of specialization, and human resource development. The findings obtained are useful for decision-making in policy planning, resource allocation, and identification of research opportunities, benefiting researchers, health professionals, and decision makers in their efforts to make significant contributions to the advancement of diabetes science.

## Introduction

### Background

The number of smartphone users worldwide has been on the rise for years. In 2021, there were 6259 million smartphone users, and this is expected to grow to 7690 million by 2027 [1]. This type of mobile technology requires specific software, commonly referred to as *apps*, according to the features and operating systems (Android or iOS) of smartphones and tablets. These apps are available for download from app stores such as Google Play Store, Apple App Store, and Amazon Appstore. Worldwide, the number of available apps has experienced an upward trend over the years. By the first quarter of 2022, the Google Play Store had approximately 3.3 million available apps, whereas Apple’s App Store had approximately 2.11 million [2]. In 2021, a total of 230 billion mobile apps were downloaded, with >US $613 billion in revenue expected by 2025 [3]. In recent years, health and fitness apps have become increasingly popular. Between the third quarter of 2019 and the second quarter of 2020, approximately 67,748 health and fitness apps were released worldwide [4]. In the third quarter of 2020, apps in the health, fitness, and nutrition category were used by 29.4% of global internet users, making it the ninth most popular app category worldwide [5]. Health care apps improve the delivery of care in a variety of ways, such as symptom assessment, disease information, and treatment progress [6].

The widespread use of mobile technologies, including apps, smartphones, tablets, and wearables, has facilitated access to health care services. Mobile health (mHealth) is a commonly used term that refers to the use of mobile technologies in the health care sector to improve patient conditions by facilitating health care initiatives such as education, intervention, medication adherence, monitoring, and disease management [7]. In addition, mHealth aims to reduce costs, encourage healthy behaviors, and empower patients by providing anytime, anywhere solutions for patients to control and manage their diseases, overcoming geographical and temporal barriers [8]. mHealth technologies, which include apps, smart devices, and sensors, are developing rapidly, supporting patient disease management especially for chronic diseases such as obesity, cancer, heart disease, and diabetes.

Diabetes mellitus, or simply diabetes, is a chronic disease caused by an abnormal carbohydrate metabolism that results in elevated levels of glucose in the blood and urine [9]. This condition can occur because the body cannot produce enough insulin, cannot produce insulin at all, or cannot effectively use insulin. There are 3 main types of diabetes: type 1, type 2, and gestational diabetes. Currently, 10.5% of the world’s population between the ages of 20 and 79 years, approximately 537 million people, are living with diabetes [9]. This number is growing every day, so it is important to empower millions of people with diabetes through the implementation of technology solutions.

Currently, there are several mHealth solution initiatives for diabetes management to support patients in their daily activities. These mainly include guidance on diabetes control, monitoring, food and medication intake, diet and physical activity tracking, and insulin administration, among other general recommendations, alerts, and reminders. However, no research has evaluated the characteristics of scientific production on mHealth for diabetes. In this sense, this study aimed to apply a scientometric analysis through a competitive technology intelligence (CTI) approach to reveal dynamics of scientific publications on mHealth for diabetes and gain insights on who the most prominent authors, countries, institutions, and journals are and what are the most cited documents. CTI adds value to assessing the technological environment, supporting decision makers in identifying opportunities to innovate and anticipate threats that could affect an organization [10]. In addition, it is supported by a methodology that can integrate different tools and disciplines (in this case, scientometrics) to measure the progress of scientific publications considering indexing elements such as authors, affiliations, countries, journal sources, and citations. This study contributes to the decision-making process for research and development (R&D) on mHealth for diabetes, adding value to science and technology output analysis.

### CTI Approach

**Figure 1:**
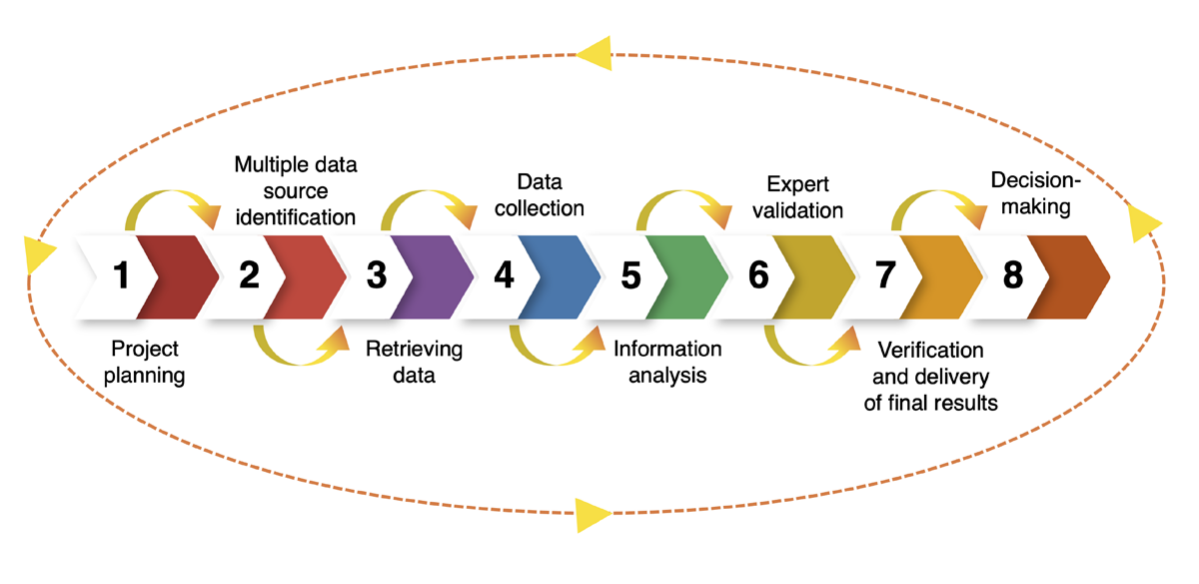
The 8-step competitive technology intelligence (CTI) methodology. Adapted from Rodriguez-Salvador and Castillo-Valdez [18].

Globalization has facilitated the flow of technology and vast amounts of information, providing opportunities to gain competitive advantages [11]. External information can influence the current and future decisions of any organization by identifying competitors, customers, suppliers [12], markets, collaborations, and disruptive events [13]. Competitive intelligence adds value to organizations in their information strategic management of the external environment [14]. The collected information should be obtained in an ethical manner [15] analyzed through an intelligence approach to identify opportunities for further strategic decisions [16]. The intelligence process involves contextualizing information, applying experience, and understanding using one’s knowledge and expertise [17]. In particular, information about the scientific and technological environment under the competitive intelligence approach is commonly known as CTI [18]. Understanding and detecting technological advances is key to identify relevant opportunities for innovation and anticipate threats from technological events [19]. CTI facilitates the technology decision-making process and helps solve the complex challenges associated with implementing or developing new advances. It includes a methodology for properly evaluating scientific and technological progress. There are different methodologies to implement the CTI approach [20], with common stages such as identification of information needs, information collection, information analysis, and information dissemination [21]. This study considered the CTI methodology proposed by Rodriguez-Salvador and Castillo-Valdez [18], which includes eight steps, as shown in Figure 1 [18]: (1) project planning, (2) multiple data source identification, (3) retrieving data, (4) data collection, (5) information analysis, (6) expert validation, (7) verification and delivery of final results, and (8) decision-making.

Initially, a planning phase is necessary to identify the needs of the organization and align them with the expected results [22], establishing development activities as well as their timelines. Subsequently, data sources need to be identified. Primary sources are based on a selection of experts, whereas secondary sources are based on reliable documents from information contained mainly in scientific databases. Selecting information involves a careful task aimed at obtaining the most relevant and emerging areas in the field of study [18]. Different types of information can be used, such as scientific literature, patents, social networks, reviews, blog posts, web-based texts, newspaper articles, and policy documents [23]. Designing a research strategy is essential when the main source of information is a scientific database; it requires appropriate queries using the core terms of interest. This is followed by data collection, where the main publications are gathered, and experts are consulted to validate the results. The integration of information should be standardized and classified to obtain a general idea of the documents’ topics and facilitate subsequent analysis. Next, in the information analysis phase of the CTI cycle, the goal is to generate intelligence [24]. In this stage, opportunities, challenges, and other critical factors are identified. In the analysis process, different data types and techniques such as scientific literature data mining, specialized patent analysis tools, and data visualization are integrated to gain valuable insights [25]. Specifically, to measure the progress of scientific literature, diverse metrics can be considered, which can be analyzed through a discipline known as scientometrics. Scientometrics aims to understand the distribution patterns of scientific literature and provides researchers with knowledge about the progress and academic impact of any field of study. It is based on a comprehensive analysis to obtain a quantitative view of research outputs, such as most prominent authors, journals, institutions, and countries [26]. The collaborative participation of experts in all steps is essential to validate the research findings through continuous feedback and corroboration of the results. Finally, the reports obtained and verified by the experts are communicated to the stakeholders to support their strategic decision-making process for R&D [18].

## Methods

In this study, the CTI methodology proposed by Rodriguez-Salvador and Castillo-Valdez [18] was considered. As was discussed in the previous section (Figure 1) [18], it consists of 8 steps in a cyclical process with continuous feedback between researchers and experts. The application of each step of this CTI methodology is explained in this section.

### Steps 1 to 2: Planning and Source Identification

The first step concerned the development of a planning process that included objectives, activities, timelines, and participants and their roles. The second step was to identify primary (experts) and secondary (databases) sources of information. For the primary sources, physicians with significant diabetes expertise from Mexico were identified to participate as experts. Particularly, Mexico ranks among the top 10 countries in the world for this illness, appearing in seventh place with 14.1 million patients with diabetes, with the highest number of adults with diabetes aged 20 to 79 years in 2021; this number is estimated to increase to 21.2 million by 2045 [9]. The selection criteria for experts included medical degrees and medical certificates from prestigious institutions, professional positions in recognized health institutions, and >10 years of experience treating patients with diabetes. A total of 3 physicians were selected, 2 practicing internal medicine and 1 practicing emergency medicine. They work in prestigious health centers in Mexico City with a high level of specialization: the Centro Medico Nacional Siglo XXI, the Instituto Nacional de Rehabilitación, and the Hospital General Regional 1 Carlos MacGregor. These experts were consulted remotely during the various stages of this CTI methodology. On the other hand, the Science Citation Index (SCI) Expanded database from Clarivate Analytics was chosen as the secondary source of information. This prestigious platform covers >9500 journals, including 182 subject categories and >61 million records [27].

### Step 3: Search Strategy and Execution

The third step involved defining the search query to be used in the SCI Expanded database to obtain the data for this study. The journal impact factor (IF) recommends searching SCI Expanded articles after the publication of the journal IF for the year of interest. In this study, the search time frame included all articles published between 1998 and 2021, meaning that the search strategy began after the announcement of the journal IF of 2021 (IF_2021_). The IF_2021_ was reported in *Journal Citation Reports* (*JCR*) on June 30, 2021. Designing an appropriate query also required identifying appropriate keywords. To this end, terms related to diabetes and mHealth technologies were identified. Quotation marks (“”) and the Boolean operator *or* were used to ensure that at least one search keyword appeared in the Topic feature of the SCI Expanded platform. This feature allows for searches of each record in the following sections: title; abstract; keywords named as author keywords; and keywords plus, which includes additional search terms retrieved from the titles of articles cited by the researcher in references and footnotes. All search keywords used in this study were found in the SCI Expanded database. In addition, the results were refined using the Article feature that this database provides in the Document Type option, which includes novel research considered citable that was, for example, published in a journal or presented at a symposium or conference [27]. The identified terms associated with diabetes and mHealth technologies were incorporated into a search query that was validated by experts, and the resulting publications were manually examined to determine whether they were relevant to the mHealth for diabetes topic. Exclusion terms were included to reduce the number of irrelevant publications. Terms associated with *app* that referred to proteins and genes and did not refer to mobile apps were excluded. In addition, terms associated with *tablet* but used for a drug rather than an electronic device were discarded. Terms associated with *wearables* that did not use mobile or wireless technology and terms associated with the materials of the wearables that did not indicate their functionality for smart devices were also excluded. Finally, terms associated with other diseases that superficially mentioned diabetes and terms associated with mHealth as a physical entity rather than mobile technology were excluded. Considering the identified exclusion keywords, the search strategy for this study can be consulted in Multimedia Appendix 1.

A total of 1848 articles from 1998 to 2021 were found in the SCI Expanded database. As Keywords Plus adds keywords that are indexed according to the Institute for Scientific Information database, also known as Clarivate Analytics [28], some publications can be irrelevant to the topic being searched [29]. To prevent this, the research group of Wang and Ho [30] was the first to propose the application of a “front page” filter that includes article titles, author keywords, and abstracts. This filter can prevent the introduction of irrelevant articles for further analysis in the scientometric study [31]. A total of 90.26% (1668/1848) of articles that included search keywords on their front page were found. Finally, the 1668 articles were manually reviewed by the authors, and a total of 1574 (94.36%) articles on mHealth for diabetes research were kept.

### Step 4 to 5: Data Collection and Information Analysis

The fourth step involved the collection of data extracted from the previously described research strategy. Data from the 1574 articles identified on mHealth for diabetes research were extracted on September 24, 2022. The full record in SCI Expanded and the number of citations each year for each publication were downloaded into Microsoft Excel 365 (Microsoft Corp), and additional coding was performed manually [32]. Diverse functions in Microsoft Excel 365 were applied, such as CONCATENATE, COUNTA, FILTER, freeze panes, LEN, MATCH, PROPER, RANK, REPLACE, SORT, SUM, and VLOOKUP.

The fifth step concerned information analysis, where the extensive scientific literature obtained was analyzed applying scientometrics. The metrics used included the number of publications and their rate by year, highly cited articles, highly cited authors, and institutions and countries with the most publications, among others. This study focused on seven main topics for the scientometric analysis: (1) publication language, (2) publication output characteristics, (3) Web of Science categories and journals, (4) country and institution publication performance, (5) author publication performance, (6) citation history of the 10 most frequently cited articles, and (7) top 10 articles with the highest impact. As mentioned previously, the journal IFs (IF_2021_) were obtained following *JCR* for the year 2021. For articles with multiple corresponding authors, all the corresponding authors, institutes, and countries were considered. Articles with corresponding authors who had only the address and no affiliation names were completed by inserting the address into the affiliation name. Author affiliations were also homogenized; for example, England, Scotland, Northern Ireland, and Wales were considered as 1 group under the United Kingdom [33]. Similarly, Harvard Medical School, United States, and Harvard University, United States, were also regrouped under Harvard University, United States. For this research, the *institutions* indicator considered universities or research institutions but not departments or research centers in a university.

Publications were assessed using the following citation indicators: (1) the number of citations from the Web of Science Core Collection in a specific year (C_year_; eg, C_2021_ describes the citation count in 2021 [34]); (2) the total number of citations received in the Web of Science Core Collection from the year of publication to the end of a specific year (TC_year_), which in this study was 2021 (TC_2021_) [35]; (3) the average number of citations per publication (CPP_year_; CPP_2021_=TC_2021_/total number of articles [TP]) [36]; and (4) average number of authors per article (APP).

In addition, six publication indicators were applied to evaluate the publication performance of countries and institutions [37]: (1) TP, (2) number of single-country articles (IP_C_) or single-institution articles (IP_I_; this indicator provides information about articles written by authors from the same country or institution), (3) number of international collaborative articles (CP_C_) or number of interinstitutional collaborative articles (CP_I_; this indicator shows whether the article was written by authors from different countries or different institutions. The institutions can be in the same country or different countries), (4) number of first-author articles (FP), (5) number of corresponding-author articles (RP), and (6) number of single-author articles (SP).

Furthermore, six citation indicators related to the 6 publication indicators (CPP_2021_) were also used to evaluate the publication impact on countries and institutions [38]: (1) the *Y*-index was used to evaluate the publication performance of authors, which is defined as *Y*-index (*j*, *h*) [34,39], where *j* is a constant related to the publication potential, the sum of the first-author articles, and the corresponding-author articles, and *h* is a constant related to the publication characteristics, the polar angle of the proportion of RP to FP (the larger the value of *j*, the more the first author and corresponding author contribute to the articles); (2) *h*=π/2 indicates that an author has only published articles as corresponding author (using this value in the *Y*-index, *j* is the RP); (3) π/2>*h*>π/4 denotes that an author has more corresponding-author articles than first-author articles (FP>0); (4) *h*=π/4 indicates that an author has the same FP and RP (FP>0 and RP>0); (5) π/4>*h*>0 indicates an author with more first-author articles than corresponding-author articles (RP>0); and (6) *h*=0 denotes that an author has only published first-author articles (using this value in the *Y*-index, *j* is the FP).

In addition, the most common author keywords were identified to determine current research hot spots. Author keywords reveal the focus that authors transmit to readers. A statistical analysis of the frequency of author keywords in the 1574 documents selected was performed using Microsoft Excel 365 through the functions COUNTA, CONCATENATE, MATCH, VLOOKUP, PROPER, RANK, REPLACE, freeze panes, SORT, SUM, and LEN. Terms related to *diabetes* and *mHealth* were discarded because all articles collected contained these; otherwise, we could erroneously conclude that, for example, the term *diabetes mellitus* was one of the main research topics in diabetes mHealth.

Charts, figures, and tables were created to present the findings of the scientometric analysis, which are described in the Results and Discussion sections.

### Step 6 to 8: Expert Validation, Delivery of Results, and Decision-Making

The sixth step of the CTI methodology was the validation of the results obtained, where experts provided valuable feedback during all steps. The seventh step allowed for the integration of the outcomes, which are presented in the Results and Discussion sections. Finally, the eighth step aimed to support the stakeholders in their decision-making process, thus promoting innovation.

## Results

### Publication Language

A total of 1574 mHealth for diabetes–related articles were obtained in 7 different languages. The most common language was English with 98.41% (1549/1574) of the articles followed by German with 1.27% (20/1574) of the articles. A total of 0.06% (1/1574) of the articles were published in French, Hungarian, Korean, and Spanish each. In addition, 0.06% (1/1574) of the articles were published in *Trials*, a bilingual (English and Esperanto) journal. Non–English-language articles had fewer citations, with a CPP_2021_ of 1.3 (SD 1.9), while English-language articles had a CPP_2021_ of 16 (SD 38). Non–English-language articles had a lower APP of 3.3 (SD 2.8), whereas English-language articles had an APP of 6.6 (SD 4.4).

### Publication Output Characteristics

**Figure 2:**
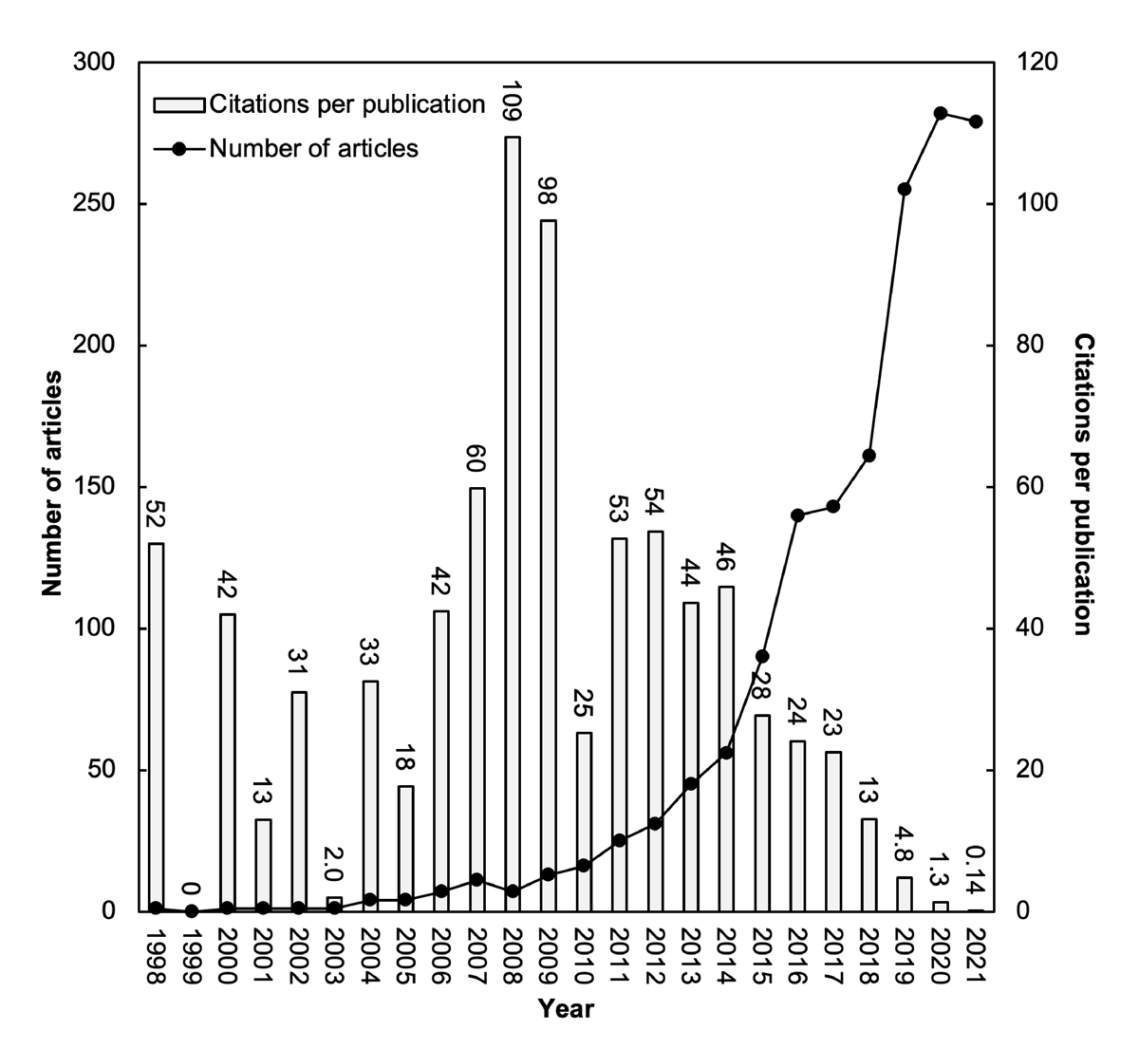
Number of articles and citations per publication by year.

To understand the trends and impacts of publications on a research topic, Ho [36] proposed a correlation between the annual number of articles (TP) and their CPP by year, which, in the last decade, has been widely applied to several medical-related topics, including dengue [40], Ebola [41], breast reconstruction [42], fracture nonunion [43], keloids [44], and Q fever [45]. Between 1998 and 2021, a total of 1574 articles associated with mHealth for diabetes were published in SCI Expanded. The mean TC_2021_ value was 15, with 705 as the maximum value for an article. The distribution of the annual number of articles and their CPP_2021_ by year is shown in Figure 2. From 1998 to 2008, the number of publications per year ranged from 1 to 11 articles except for 1999, when no articles were published. There was an increase from 7 articles in 2008 to 56 articles in 2014, a total of 282 articles in 2020, and 279 articles in 2021. In 2008, a total of 7 articles with a TC_2021_ of 766 had the highest CPP_2021_ at 109, followed by a CPP_2021_ of 98 in 2009. The second most frequently cited article, titled “Healthcare via Cell Phones: A Systematic Review” [46], in mHealth for diabetes research was published in 2009 with a TC_2021_ of 550. Similarly, in 2008, an article titled “WellDoc (TM) Mobile Diabetes Management Randomized Controlled Trial: Change in Clinical and Behavioral Outcomes And Patient And Physician Satisfaction” [47] ranked ninth with a TC_2021_ of 212. On the basis of Figure 2, it takes approximately 8 years for the CPP to reach a plateau. It took a shorter time to reach a plateau than for other medical topics, such as fracture nonunion (14 years) [43] and breast reconstruction (10 years) [42].

### Web of Science Categories

In 2021, *JCR* indexed 9649 journals in all areas with citation references across 178 Web of Science categories in SCI Expanded. For this study, the Web of Science categories were identified based on their CPP_year_ and APP as basic information [38,43]. By 2021, a total of 491 journals published articles related to mHealth for diabetes in 92 Web of Science categories in SCI Expanded. Table 1 shows the top 10 most productive categories, mainly Health Care Sciences & Services (109 journals), Medical Informatics (31 journals), and Endocrinology & Metabolism (148 journals). Comparing the top 10 categories, articles published in the Endocrinology & Metabolism category had the highest CPP_2021_ at 21, whereas Engineering, Electrical & Electronic articles had a CPP_2021_ of 6.7. Articles published in the Medicine, General & Internal category had the highest APP at 7.8.

**Table 1:** Top 10 most productive Web of Science categories (N=1574).

Web of Science category	TP^a^, n (%)	Journals in each category, n	AU^b^	APP^c^ (SD)	TC_2021_^d^	CPP_2021_^e^ (SD)
Health Care Sciences & Services	409 (26)	109	2548	6.2 (4.7)	6952	17 (39)
Medical Informatics	362 (23)	31	2323	6.4 (4.7)	4961	14 (28)
Endocrinology & Metabolism	262 (16.6)	148	1937	7.4 (4.9)	5497	21 (39)
Computer Science, Information Systems	117 (7.4)	164	689	5.9 (7.0)	1647	14 (24)
Public, Environmental & Occupational Health	114 (7.2)	210	778	6.8 (3.4)	1190	10 (22)
Engineering, Electrical & Electronic	108 (6.9)	278	540	5.0 (2.0)	722	6.7 (12)
Medicine, General & Internal	106 (6.7)	172	826	7.8 (5.1)	1677	16 (43)
Chemistry, Analytical	98 (6.2)	87	525	5.4 (2.5)	1394	14 (28)
Instruments & Instrumentation	69 (4.4)	64	370	5.4 (2.7)	587	8.5 (22)
Computer Science, Interdisciplinary Applications	62 (3.9)	113	346	5.6 (2.7)	653	11 (14)

^a^TP: total number of articles.

^b^AU: number of authors in a category.

^c^APP: average number of authors per publication.

^d^TC_2021_: total number of citations from the Web of Science Core Collection from the publication year to the end of 2021.

^e^CPP_2021_: average number of citations per publication.

**Figure 3:**
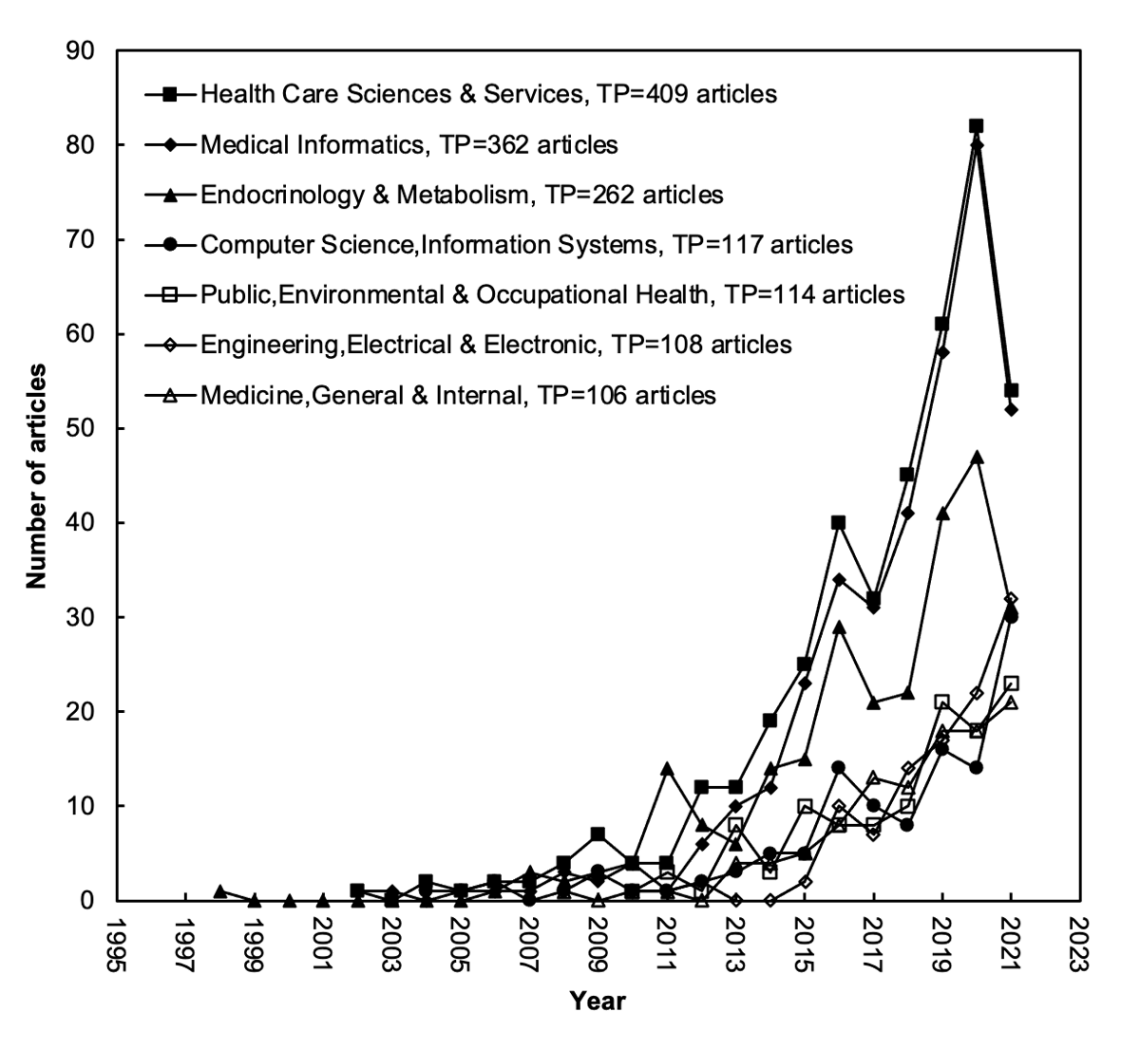
Development trends of the top 7 Web of Science categories with a total number of articles (TP) of >100.

The evolution of the top 7 Web of Science categories with >100 articles is illustrated in Figure 3. The Health Care Sciences & Services and Medical Informatics categories have shown a similar trend since 2002. From 2010 to 2021, a total of 108 articles were published in the Engineering, Electrical & Electronic category (ranked sixth), with 32 articles published in 2021. The first article on mHealth for diabetes was published in the Endocrinology & Metabolism category.

### Journals

Recently, Ho [48] proposed to measure the characteristics of journals according to their CPP_year_ and APP. Table 2 presents the top 10 most productive journals in terms of journal IFs, CPP_2021_, and APP. *JMIR mHealth and uHealth* (IF_2021_=4.947) published the highest number of articles, in this case, 7.94% (125/1574). Comparing the top 10 most productive journals, mHealth for diabetes articles published in *Diabetes Care* (IF_2021_=17.152) had the highest CPP_2021_ at 59, whereas articles published in *BMJ Open* (IF_2021_=3.006) had a CPP_2021_ of only 2.8. The APP ranged from 12 in *Diabetes Care* to 4.8 in the *Journal of Telemedicine and Telecare* and *Telemedicine and e-Health*. According to the IF_2021_, the top 3 journals with an IF_2021_ of >100 were *The* *Lancet* (IF_2021_=202.731) with 1 article, the *New England Journal of Medicine* (IF_2021_=176.079) with 1 article, and the *Journal of the American Medical Association* (IF_2021_=157.335) with 1 article. These 3 journals were also the top 3 journals among 172 journals in the Web of Science category of General and Internal Medicine.

**Table 2:** Top 10 most productive journals (N=1574).

Journal	TP^a^, n (%)	IF_2021_^b^	APP^c^ (SD)	CPP_2021_^d^ (SD)	Web of Science category
*JMIR mHealth and uHealth*	125 (7.9)	4.947	6.3 (2.7)	10 (19)	Health Care Sciences & Services Medical Informatics
*Journal of Medical Internet Research*	92 (5.8)	7.076	7.0 (3.3)	21 (41)	Health Care Sciences & Services Medical Informatics
*Diabetes Technology & Therapeutics*	69 (4.4)	7.337	7.2 (4.5)	19 (36)	Endocrinology & Metabolism
*Sensors*	34 (2.2)	3.847	5.2 (2.1)	7.3 (10)	Chemistry, Analytical Engineering, Electrical & Electronic Instruments & Instrumentation
*BMJ Open*	32 (2)	3.006	12 (6.5)	2.8 (3.6)	Medicine, General & Internal
*Journal of Telemedicine and Telecare*	30 (1.9)	6.344	4.8 (1.9)	28 (31)	Health Care Sciences & Services
*Telemedicine and e-Health*	30 (1.9)	5.033	4.8 (2.3)	21 (27)	Health Care Sciences & Services
*Diabetes Care*	27 (1.7)	17.152	12 (7.2)	59 (61)	Endocrinology & Metabolism
*PLOS One*	26 (1.7)	3.752	5.7 (2.3)	24 (34)	Multidisciplinary Sciences
*Trials*	24 (1.5)	2.728	10 (3.4)	6.4 (8.6)	Medicine, Research & Experimental

^a^TP: total number of articles.

^b^IF_2021_: journal impact factor in 2021.

^c^APP: average number of authors per article.

^d^CPP_2021_: average number of citations per paper (total number of citations/TP).

### Country and Institution Publication Performance

It is widely recognized that the first author and the corresponding author are the 2 authors who contribute the most to a research article [49]. At the institutional level, the affiliation specified for the corresponding author might be where they are based or the origin of the article [34]. In this study, a total of 1574 articles were published by authors from 90 countries, 1129 (71.73%) of which were single-country articles with authors from 62 countries and a CPP_2021_ of 15, whereas 445 (28.27%) were international collaborative articles with authors from 83 countries and a CPP_2021_ of 16. The results showed that international collaborative articles slightly increased citations in mHealth for diabetes research. The 6 publication indicators and the 6 related citation indicators (CPP_2021_) [38] described in the Methods section were applied to determine the top 10 most productive countries, which are presented in Table 3. The United States ranked first in 5 of the 6 publication indicators, with a TP of 566 articles, representing 35.96% (566/1574). These publication indicators were an IP_C_ of 367 articles, corresponding to 32.51% (367/1129) of the single-country articles; a CP_C_ of 199 articles, which corresponds to 44.7% (199/445) of the international collaborative articles; an FP of 453 articles, which constitutes 28.78% (453/1574) of the first-author articles; and an RP of 465 articles, equating to 29.54% (465/1574) of the corresponding-author articles. Meanwhile, Germany ranked first in the SP with 8 articles, representing 22% (8/36) of the single-author articles. Among the top 10 most productive countries, South Korea had a TP of 95 articles, an IP_C_ of 67 articles, a CP_C_ of 28 articles, an FP of 82 articles, an RP of 85 articles, and an SP of 2 articles. Nevertheless, South Korea had the highest TP-CPP_2021_, IP_C_-CPP_2021_, CP_C_-CPP_2021_, FP-CPP_2021_, RP-CPP_2021_, and SP-CPP_2021_, with values of 35, 32, 40, 37, 36, and 61, respectively.

**Table 3:** Top 10 most productive countries.

Country	TP^a^, n (%)	TP		IP_C_^b^			CP_C_^c^			FP^d^			RP^e^			SP^f^	
		Rank	CPP_2021_^g^ (SD)	Rank (%)^h^	CPP_2021_ (SD)	Rank (%)^i^		CPP_2021_ (SD)	Rank (%)^j^		CPP_2021_ (SD)	Rank (%)^k^		CPP_2021_ (SD)	Rank (%)^l^		CPP_2021_ (SD)
United States	566 (35.96)	1	22 (52)	1 (33)	21 (47)	1 (45)		24 (59)	1 (29)		20 (44)	1 (30)		21 (44)	2 (11)		2.8 (2.8)
United Kingdom	172 (10.93)	2	13 (24)	4 (5.2)	15 (28)	2 (25)		12 (22)	3 (6.4)		14 (25)	3 (7.1)		14 (24)	10 (2.8)		0 (n)
China	137 (8.70)	3	11 (28)	2 (7.4)	6.7 (13)	4 (12)		18 (42)	2 (7.6)		11 (30)	2 (7.7)		11 (20)	4 (8.3)		29 (29)
Australia	106 (6.73)	4	10 (20)	5 (4.1)	12 (26)	3 (13)		8.1 (13)	5 (4.3)		11 (22)	5 (4.4)		11 (22)	—^m^		—
South Korea	95 (6.04)	5	35 (85)	3 (5.9)	32 (57)	11 (6.3)		40 (132)	4 (5.2)		37 (91)	4 (5.4)		36 (90)	6 (5.6)		61 (34)
Germany	79 (5.02)	6	9.6 (24)	8 (2.7)	2.5 (4.2)	5 (11)		14 (29)	6 (3.7)		11 (27)	6 (3.7)		8.5 (19)	1 (22)		1.0 (1.9)
India	73 (4.64)	7	13 (26)	7 (3.2)	11 (22)	8 (8.3)		15 (29)	6 (3.7)		13 (27)	6 (3.7)		13 (27)	—		—
Spain	72 (4.57)	8	10 (16)	6 (3.7)	11 (19)	10 (6.7)		8.1 (11)	8 (3.3)		11 (18)	8 (3.5)		10 (18)	—		—
Canada	70 (4.45)	9	21 (45)	9 (2.7)	23 (55)	6 (9.0)		19 (36)	9 (2.9)		26 (53)	9 (2.9)		26 (53)	4 (8.3)		20 (21)
The Netherlands	60 (3.81)	10	22 (29)	14 (1.8)	23 (33)	6 (9.0)		21 (26)	14 (1.7)		25 (36)	14 (1.8)		24 (35)	—		—

^a^TP: total number of articles and percentage of the total number of mobile health for diabetes articles (N=1574).

^b^IP_C_: number of single-country articles.

^c^CP_C_: number of international collaborative articles.

^d^FP: number of first-author articles.

^e^RP: number of corresponding-author articles.

^f^SP: number of single-author articles.

^g^CPP_2021_: average number of citations per publication (total number of citations/TP).

^h^Rank and percentage of all single-country articles.

^i^Rank and percentage of all international collaborative articles.

^j^Rank and percentage of all first-author articles.

^k^Rank and percentage of all corresponding-author articles.

^l^Rank and percentage of all single-author articles.

^m^Not available.

**Figure 4:**
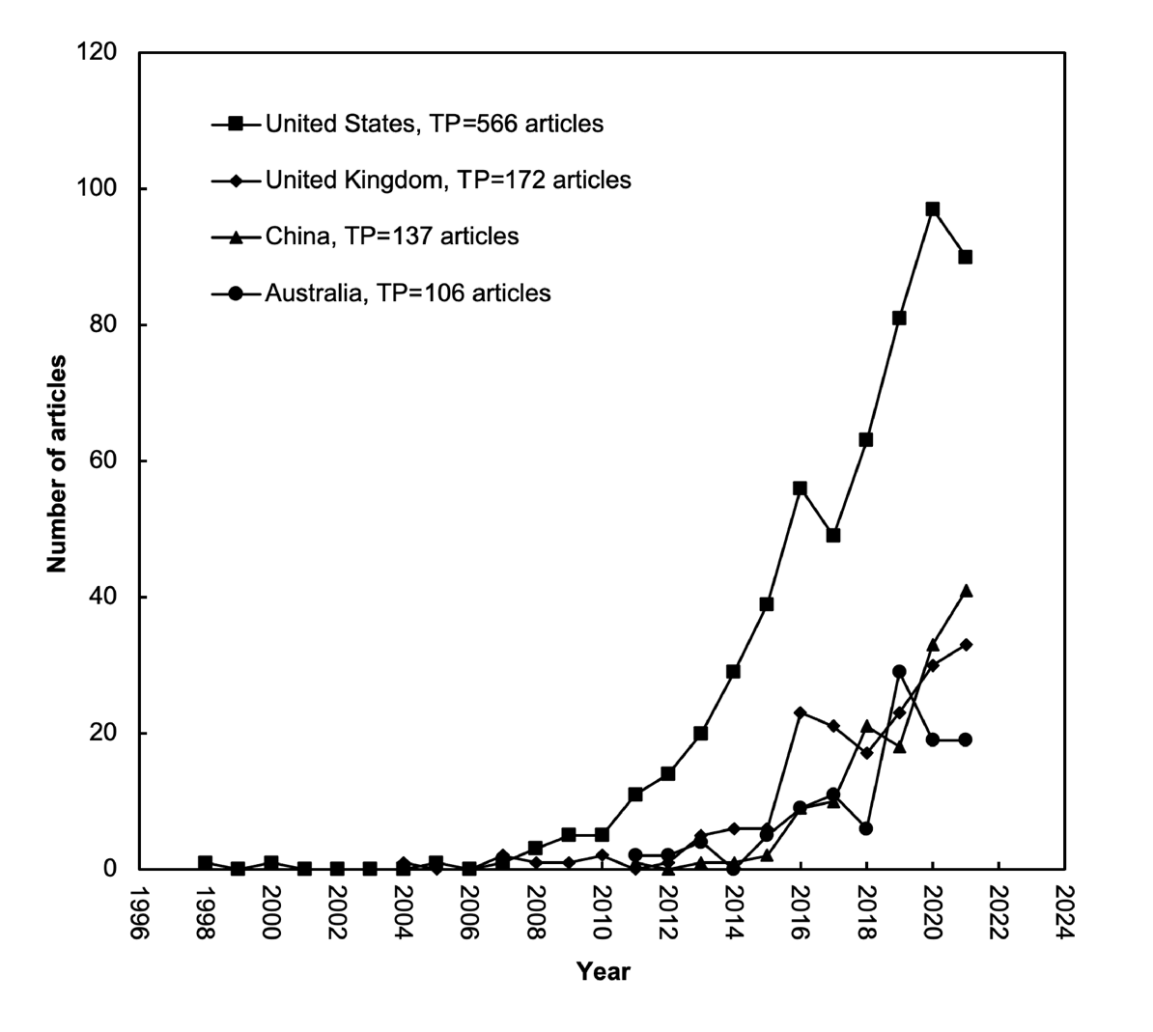
Development trends of the top 4 countries with a total number of articles (TP) of >100. UK: United Kingdom; USA: United States of America.

Publication trends for the top 4 most productive countries are presented in Figure 4. The United States, the United Kingdom, China, and Australia were the leaders, all with >100 articles. The only article on mHealth for diabetes in the 1990s was published by authors from the United States. Since 2008, the United States has been at the top with a significant upward trend. China and Australia each published their first article in 2011. In 2021, China reached the second position with 41 articles.

In addition, as shown in Table 4, the top 10 most productive countries in terms of publications were compared with respect to diabetes-related health expenditure.

**Table 4:** Top 10 most productive countries in terms of publications related to mobile health for diabetes and diabetes-related health expenditure (N=1574).

Country	Total number of articles^a^, n (%)	Diabetes-related health expenditure (million US $) [50]			Adults aged between 20 and 79 years with diabetes (thousands) [51]			Prevalence of diabetes in adults aged between 20 and 79 years (%) [52]		
		2021	2030	2045	2021	2030	2045	2021	2030	2045
United States	566 (36)	379,470.20	388,934.40	392,537.40	32,215.30	34,755.30	36,289.90	10.7	12.1	12.9
United Kingdom	172 (10.9)	23,415.40	23,815.40	23,949.20	3,996.30	4,140.60	4,408.10	6.3	7	7.5
China	137 (8.7)	165,304.00	185,008.20	193,142.70	140,869.60	164,069.50	174,433.50	10.6	11.8	12.5
Australia	106 (6.7)	8867.10	9588.00	10,405.70	1,491.80	1,693.00	1,935.20	6.4	7.4	8
South Korea	95 (6)	8971.20	9182.80	7807.80	3,511.80	3,934.20	3,860.90	6.8	7.2	7.8
Germany	79 (5)	41,295.80	42,364.90	37,913.80	6,199.90	6,519.70	6,094.40	6.9	7.9	8.4
India	73 (4.6)	8485.80	10,305.50	12,834.30	74,194.70	92,973.70	124,874.70	9.6	10.4	10.8
Spain	72 (4.6)	15,453.60	15,625.30	14,169.20	5,141.30	5,576.00	5,647.60	10.3	11.8	12.7
Canada	70 (4.4)	14,284.10	14,905.90	15,259.40	2,974.00	3,288.20	3,468.50	7.7	8.9	9.6
The Netherlands	60 (3.8)	5523.10	5481.10	5129.20	857	910.4	894.7	4.5	5.3	5.8

^a^Data previously presented in Table 3.

**Figure 5:**
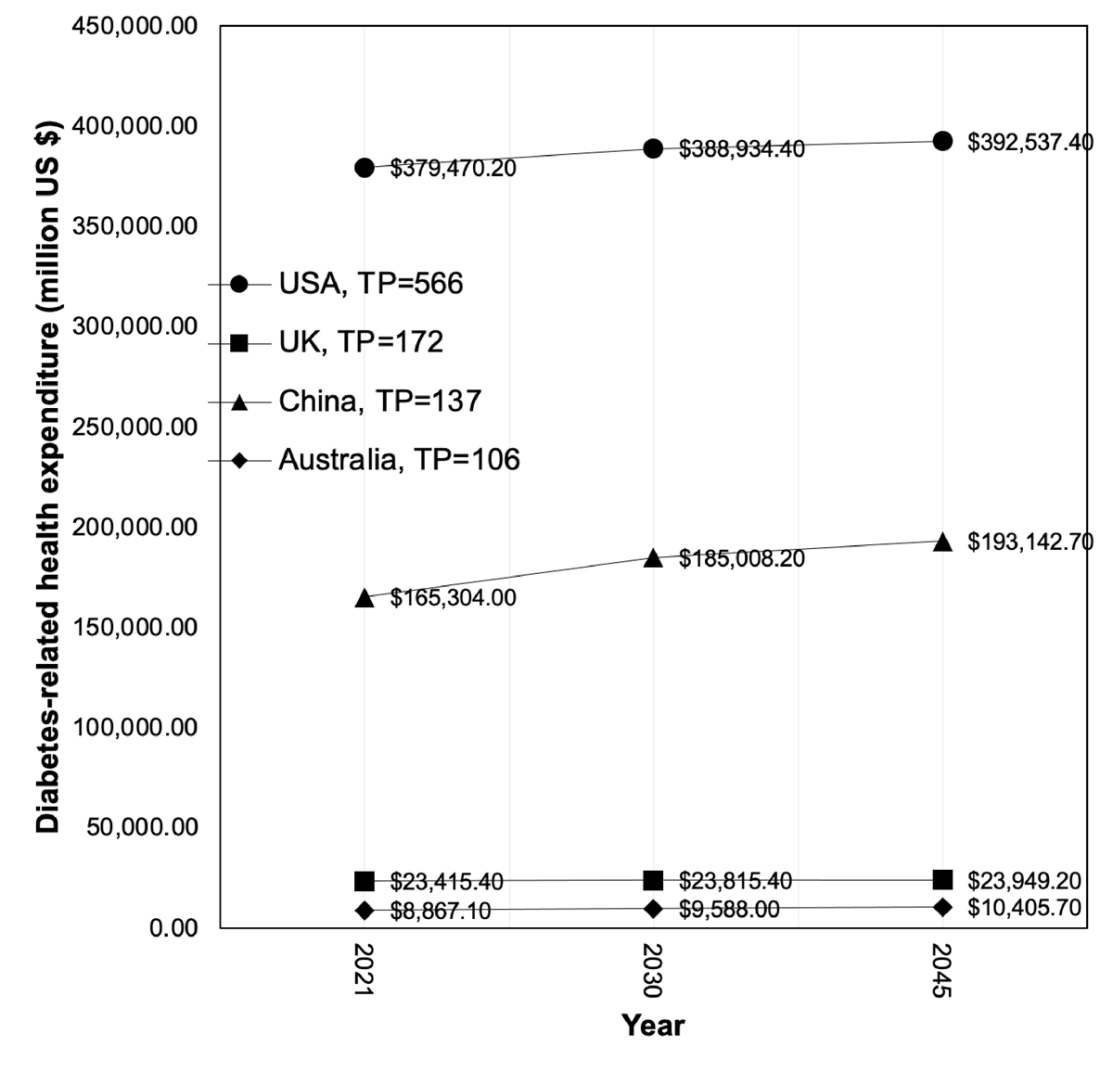
Diabetes-related health expenditure of the top 4 most productive countries in terms of publications. UK: United Kingdom; USA: United States of America.

Between 2021 and 2045, the United States, United Kingdom, China, Australia, India, and Canada will increase their diabetes-related health expenditure, whereas South Korea, Germany, Spain, and the Netherlands will decrease it. Trends in diabetes-related health expenditure of the top 4 most productive countries in terms of publications are shown in Figure 5.

Regarding institutions, 23.57% (371/1574) of the mHealth for diabetes articles came from a single institution, with a CPP_2021_ of 14, whereas 76.43% (1203/1574) of the articles were interinstitutional collaborations, with a CPP_2021_ of 16. As before, collaboration slightly increased citations in mHealth for diabetes research. The top 10 most productive institutions and their characteristics are shown in Table 5. Harvard University in the United States ranked first with a TP of 59 articles (59/1574, 3.75%), a CP_I_ of 57 articles (57/1203, 4.7% of the interinstitutional collaborative articles), and an RP of 18 articles (18/1574, 1.14% of the corresponding-author articles). The University of Michigan in the United States ranked first in first-author articles with an FP of 14 articles (14/1574, 0.89% of the first-author articles). In addition, James Cook University in Australia had a total of 6 articles (ranked 89th), all of which were single-institution articles, obtaining first place for single-institution articles with an IP_I_ of 6 articles (6/371, 1.6% of the single-institution articles). The University of Toronto in Canada is the only institution with single-author articles in Table 5. Among the top 10 most productive institutions, Seoul National University in South Korea had a TP of 20 articles, a CP_I_ of 15 articles, and an RP of 17, with the highest TP-CPP_2021_, CP_I_-CPP_2021_, and RP-CPP_2021_ at 76, 93, and 83, respectively. The University of California, San Francisco, in the United States had an IP_I_ of 4 articles, with the highest IP_I_-CPP_2021_ at 41. Meanwhile, the University of Virginia in the United States had an FP of 8 articles, with the highest FP-CPP_2021_ at 44.

**Table 5:** Top 10 most productive institutions.

Institution	TP^a^, n (%)	TP			IP_I_^b^			CP_I_^c^			FP^d^			RP^e^	
		Rank	CPP_21_^f^ (SD)	Rank (%)^g^		CPP_21_ (SD)	Rank (%)^h^		CPP_21_ (SD)	Rank (%)^i^		CPP_21_ (SD)	Rank (%)^j^		CPP_21_ (SD)
Harvard University, United States	59 (3.75)	1	22 (46)	16 (0.54)		18 (25)	1 (4.7)		23 (47)	2 (0.76)		18 (22)	1 (1.1)		13 (20)
Stanford University, United States	34 (2.16)	2	23 (41)	16 (0.54)		15 (0.71)	2 (2.7)		23 (42)	5 (0.64)		18 (23)	7 (0.70)		36 (66)
University of Michigan, United States	26 (1.65)	3	31 (39)	16 (0.54)		7.5 (11)	3 (2.0)		33 (40)	1 (0.89)		24 (22)	3 (0.89)		22 (23)
University of California, San Francisco, United States	23 (1.46)	4	21 (27)	8 (0.81)		41 (31)	5 (1.7)		19 (26)	5 (0.64)		27 (29)	4 (0.76)		22 (28)
Duke University, United States	22 (1.40)	5	15 (28)	2 (1.3)		33 (46)	7 (1.4)		9.5 (18)	5 (0.64)		18 (35)	4 (0.76)		18 (32)
University of Toronto, Canada	21 (1.33)	6	35 (72)	—^k^		—	4 (1.7)		35 (72)	48 (0.25)		37 (73)	86 (0.19)		49 (85)
Seoul National University, South Korea	20 (1.27)	7	76 (169)	2 (1.3)		25 (48)	10 (1.2)		93 (192)	2 (0.76)		19 (31)	1 (1.1)		83 (183)
University of Oxford, United Kingdom	20 (1.27)	7	12 (25)	54 (0.27)		15 (—)	6 (1.6)		12 (26)	11 (0.51)		4.1 (5.9)	11 (0.57)		6.2 (8.4)
University of California, San Diego, United States	18 (1.14)	9	16 (28)	6 (1.1)		5.5 (11)	16 (1.2)		19 (31)	8 (0.57)		17 (37)	11 (0.57)		20 (37)
University of Virginia, United States	18 (1.14)	9	51 (40)	8 (0.81)		20 (32)	10 (1.2)		57 (40)	11 (0.51)		44 (48)	27 (0.38)		48 (52)

^a^TP: total number of articles and percentage of all mobile health for diabetes articles (N=1574).

^b^IP_I_: number of single-institution articles.

^c^CP_I_: number of interinstitutional collaborative articles.

^d^FP: number of first-author articles.

^e^RP: number of corresponding-author articles.

^f^CPP_21_: average number of citations per publication (total number of citations/TP).

^g^Rank and percentage of all single-institution articles.

^h^Rank and percentage of all interinstitutional collaborative articles.

^i^Rank and percentage of all first-author articles.

^j^Rank and percentage of all corresponding-author articles.

^k^Not available.

### Author Publication Performance

The 1574 articles on mHealth for diabetes had an APP of 6.5, whereas the maximum number of authors in an article was 73. A total of 5.59% (88/1574) of the articles had multiple corresponding authors, whereas the maximum number of corresponding authors, institutions, and countries associated with an article were 11, 8, and 4, respectively. Articles published by groups of 3 to 7 authors accounted for 61.94% (975/1574) of the total. These groups consisted of 4, 6, 5, 3, and 7 authors, with 15.5% (244/1574), 13.47% (212/1574), 11.94% (188/1574), 11.05% (174/1574), and 9.97% (157/1574) of the articles, respectively. Table 6 lists the top 20 most productive authors in mHealth for diabetes research considering 4 publication indicators, their corresponding citation indicators, and *Y*-index constants. E Arsand published the most articles with 18. HS Kim published the most first-author articles with 7 and the most corresponding-author articles with 10. Kim was the only author in the top 20 who had single-author articles. Comparing the top 20 most productive authors, A Farret had a TP of 9 articles, with the highest TP-CPP_2021_ at 77. CC Quinn had an FP of 7 articles and an RP of 7 articles, with the highest FP-CPP_2021_ at 83 and the highest RP-CPP_2021_ at 83. Only 5 of the top 20 most productive authors, namely, HS Kim, CC Quinn, CG Parkin, JD Piette, and J Wang, were among the top 20 publication potential authors according to the *Y*-index.

**Figure 6:**
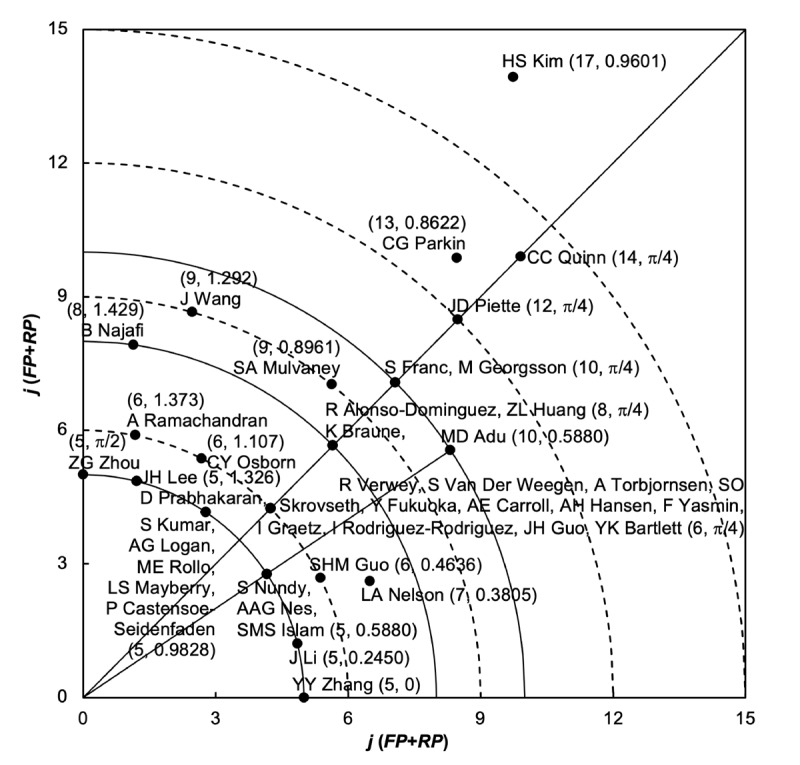
Top 43 authors with a Y-index of (j≥5). FP: number of first-author articles; RP: number of corresponding-author articles.

Authors were extensively studied based on the *Y*-index. The 1574 articles on mHealth for diabetes were produced by 7922 authors. Of these 7922 authors, 5975 (75.42%) had no first-author articles and no corresponding-author articles, with a *Y*-index of (0, 0); 596 (7.52%) published only corresponding-author articles, with *h*=π/2; 512 (6.46%) published more corresponding-author articles than first-author articles, with π/2>*h*>π/4 (FP>0); 263 (3.32%) published the same number of first-author and corresponding-author articles, with *h*=π/4 (FP>0 and RP>0); 25 (0.32%) published more first-author articles than corresponding-author articles, with π/4>*h*>0 (RP>0); and 551 (6.96%) published only first-author articles, with *h*=0. The polar coordinates are shown in Figure 6, which illustrates the distribution of the *Y*-index (*j*, *h*) of the leading 43 publication potential authors in mHealth for diabetes research with *j*≥5. Each point has a coordinate *Y*-index (*j*, *h*), which represents the publication performance of the authors. In some cases, authors can coincide at the same point; for example, R Verwey, S Van Der Weegen, A Torbjornsen, SO Skrovseth, YJH Guo, Fukuoka, AE Carroll, AH Hansen, F Yasmin, I Graetz, I Rodriguez-Rodriguez, and YK Bartlett had a *Y*-index of (6, p/4). The greatest publication potential was found for HS Kim, with a *Y*-index of (17, 0.9601) and a *j* of 17, followed by CC Quinn with a *Y*-index of (14, π/4), CG Parkin with a *Y*-index of (13, 0.8622), and JD Piette with a *Y*-index of (12, π/4).

**Table 6:** Top 20 most productive authors with ≥9 articles.

Author	TP^a^		FP^b^			RP^c^			SP^d^			*h* ^e^		Rank (*j* ^f^)
	Rank (TP)	CPP_2021_^g^ (SD)	Rank (FP)	CPP_2021_ (SD)	Rank (RP)		CPP_2021_ (SD)	Rank (SP)		CPP_2021_ (SD)				
E Arsand	1 (18)	29 (29)	157 (1)	51 (—^h^)	55 (2)		70 (26)	—		—	1.107		113 (3)	
HS Kim	2 (15)	40 (44)	1 (7)	49 (29)	1 (10)		58 (43)	1 (2)		61 (34)	0.9601		1 (17)	
X Li	3 (13)	8.3 (20)	—	—	196 (1)		0 (—)	—		—	p/2		910 (1)	
E Renard	4 (12)	59 (40)	35 (2)	57 (3.5)	55 (2)		57 (3.5)	—		—	p/4		44 (4)	
S Del Favero	4 (12)	58 (42)	18 (3)	60 (4.7)	196 (1)		6.0 (—)	—		—	0.3218		44 (4)	
C Cobelli	6 (11)	63 (41)	—	—	12 (4)		51 (18)	—		—	p/2		44 (4)	
E Dassau	6 (11)	32 (39)	—	—	12 (4)		18 (15)	—		—	p/2		44 (4)	
J Wang	6 (11)	16 (33)	35 (2)	13 (11)	2 (7)		22 (41)	—		—	1.292		8 (9)	
N Tandon	6 (11)	13 (21)	—	—	55 (2)		14 (16)	—		—	p/2		169 (2)	
D Bruttomesso	10 (10)	71 (34)	—	—	—		—	—		—	0		1948 (0)	
FJ Doyle	10 (10)	37 (43)	—	—	—		—	—		—	0		1948 (0)	
J Li	10 (10)	10 (16)	10 (4)	1.8 (3.5)	196 (1)		3.0 (—)	—		—	0.2450		31 (5)	
A Avogaro	13 (9)	75 (34)	—	—	—		—	—		—	0		1948 (0)	
A Farret	13 (9)	77 (48)	—	—	—		—	—		—	0		1948 (0)	
CC Quinn	13 (9)	68 (105)	1 (7)	83 (116)	2 (7)		83 (116)	—		—	p/4		2 (14)	
CG Parkin	13 (9)	1.7 (3.9)	3 (6)	0.50 (0.84)	2 (7)		2.1 (4.4)	—		—	0.8622		3 (13)	
DG Armstrong	13 (9)	14 (12)	157 (1)	14 (—)	196 (1)		8.0 (—)	—		—	p/4		169 (2)	
J Car	13 (9)	15 (25)	—	—	—		—	—		—	0		1948 (0)	
JD Piette	13 (9)	39 (32)	3 (6)	48 (35)	6 (6)		48 (35)	—		—	p/4		4 (12)	
P Keith-Hynes	13 (9)	72 (37)	—	—	—		—	—		—	0		1948 (0)	

^a^TP: total number of articles.

^b^FP: first-author articles.

^c^RP: corresponding-author articles.

^d^SP: single-author articles.

^e^A *Y*-index constant related to the publication characteristics.

^f^A *Y*-index constant related to the publication potential.

^g^CPP_2021_: average number of citations per publication (total number of citations/TP).

^h^Not available.

ZG Zhou (5, π/2), D Prabhakaran (5, 1.326), JH Lee (5, 1.326), LS Mayberry (5, 0.9828), S Kumar (5, 0.9828), AG Logan (5, 0.9828), ME Rollo (5, 0.9828), P Castensoe-Seidenfaden (5, 0.9828), SMS Islam (5, 0.5880), AAG Nes (5, 0.5880), S Nundy (5, 0.5880), J Li (5, 0.245), and YY Zhang (5, 0) all had the same *j* of 5. All these authors are located on the same curve (*j*=5) shown in Figure 6, indicating that they had the same publication potential in mHealth for diabetes research but different publication characteristics [53]. ZG Zhou published only corresponding-author articles, with an *h* of p/2. Prabhakaran and Lee had a higher ratio of corresponding-author articles to first-author articles, with an *h* of 1.326, than LS Mayberry, S Kumar, AG Logan, ME Rollo, and P Castensoe-Seidenfaden, with an *h* of 0.9828. The ratio of first-author articles to corresponding-author articles was higher for J Li with an *h* of 0.245 than for SMS Islam, AAG Nes, and S Nundy, who had an *h* of 0.5880. YY Zhang published only first-author articles, with an *h* of 0.

A Ramachandran (6, 1.373), CY Osborn (6, 1.107), R Verwey (6, π/4), and the 11 other authors, as well as SHM Guo (6, 0.4636), are on the same curve (*j*=6) with the same publication potential. However, Ramachandran had a higher ratio of corresponding-author articles to first-author articles with an *h* of 1.373, followed by Osborn with an *h* of 1.107, whereas Verwey and the other 11 authors published the same number of first-author and corresponding-author articles, with an *h* of π/4, and Guo published more first-author articles than corresponding-author articles. Similar behavior was found for authors located at a *j* of 8 and 9. MD Adu (10, 0.5880), S Nundy (5, 0.5880), AAG Nes (5, 0.5880), and SMS Islam (5, 0.5880) lie on a straight line (*h*=0.5880), indicating that they had the same publication characteristics but different publication potentials. Adu had a higher publication potential (with a *j* of 10) than Nundy, Nes, and Islam, who had a *j* of 5. Similar situations for authors located on an *h* of p/4 (the diagonal line) indicate that the authors had the same publication characteristics but different publication potentials. The location on the graph along the curves represents the publication potential, whereas the location along a straight line from the origin represents the publication characteristics. When authors are located on the same curves, this means that they have the same publication potential, whereas when authors are located on the same straight line from the origin, this means that they have the same publication characteristics.

### Citation History of the 10 Most Frequently Cited Articles

**Figure 7:**
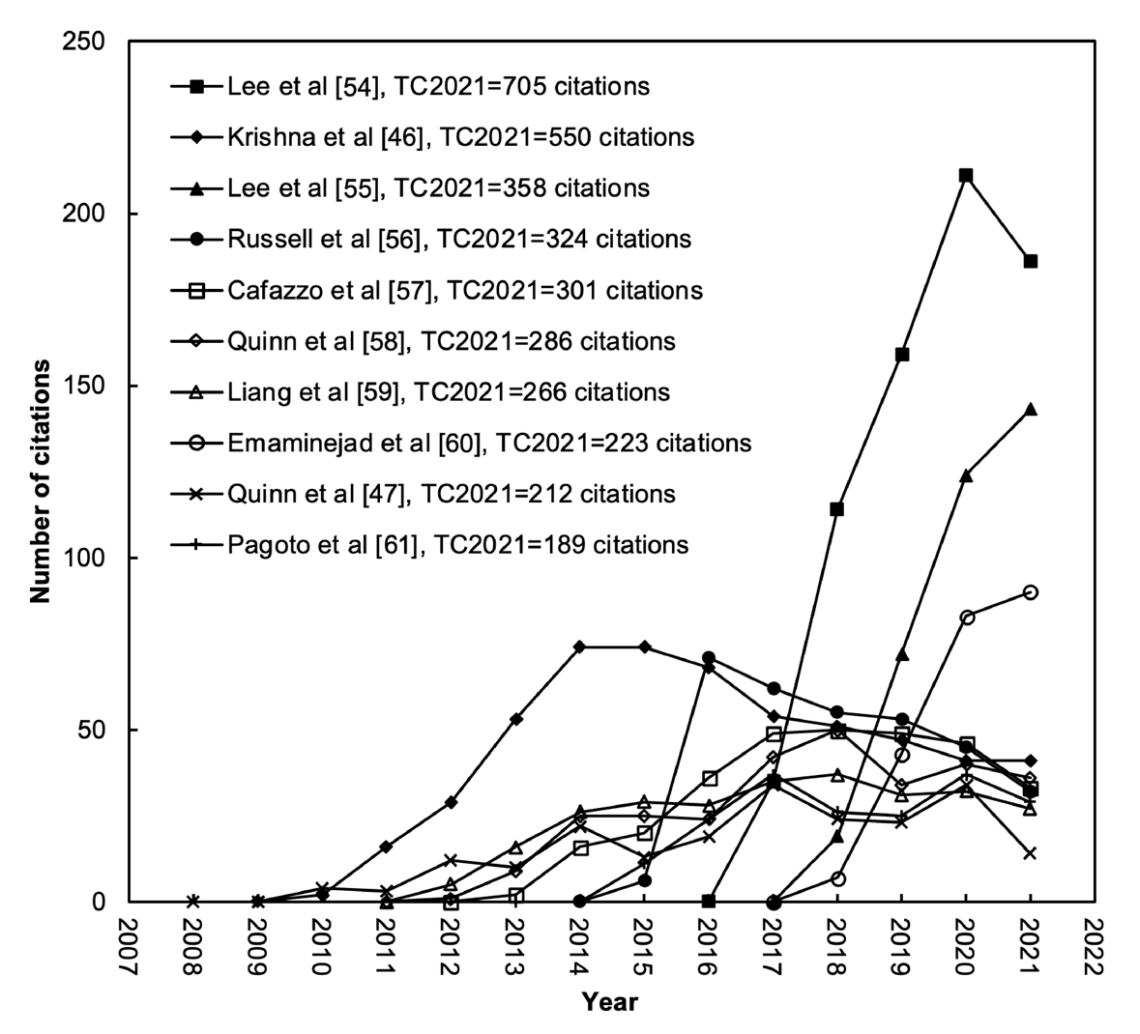
Citation history of the top 10 most frequently cited articles [46,47,51-58]. TC2021: total number of citations from the year of publication to the end of 2021.

The citations of a highly cited article are not always high [39]. It is necessary to understand the citation history of a highly cited article in an area of research. The citation history of the top 10 most frequently cited articles on mHealth for Diabetes is presented in Figure 7 [46,47,54-61]. The article by Lee et al [54] published in 2016 titled “A Graphene-Based Electrochemical Device With Thermoresponsive Microneedles for Diabetes Monitoring and Therapy” was the most frequently cited with a TC_2021_ of 705 (ranked first). The 2009 article by Krishna et al [46] titled “Healthcare via Cell Phones: A Systematic Review” had a high impact from 2011 to 2015 with a TC_2021_ of 550 (ranked second). The 2017 article by Lee et al [55] titled “Wearable/Disposable Sweat-Based Glucose Monitoring Device With Multistage Transdermal Drug Delivery Module” had a similar trend with a TC_2021_ of 358 (ranked third). The rest of the top 10 most frequently cited articles were those by Russell et al [56] (ranked 4th), Cafazzo et al [57] (ranked 5th), Quinn et al [58] (ranked 6th), Liang et al [59] (ranked 7th), Emaminejad et al [60] (ranked 8th), Quinn et al [47] (ranked 9th), and Pagoto et al [61] (ranked 10th).

### Top 10 Articles With the Highest Impact

The top 10 articles with the highest impact in 2021 are shown in Table 7. These articles had a C_2021_ of ≥46. The top 10 articles were published by 96 authors from 30 institutions in the United States, South Korea, India, Australia, and Germany. The United States published 5 of the top 10 articles. The Institute for Basic Science and Seoul National University in South Korea published 3 of the top 10 articles. The top 3 articles were ranked in both the top 10 most frequently cited articles and the top 10 articles with the highest impact in 2021. These 3 highly cited and most impactful articles are summarized in the following paragraphs, as well as the articles that ranked fourth and fifth.

**Table 7:** The top 10 articles with the highest impact in 2021 with a number of citations in 2021 (C2021) of ≥46.

Rank: C_2021_	Rank: TC_2021_^a^	Title	Country	Reference
1 (186)	1 (705)	“A Graphene-Based Electrochemical Device With Thermoresponsive Microneedles for Diabetes Monitoring and Therapy”	South Korea and the United States	Lee et al [54]
2 (143)	3 (358)	“Wearable/Disposable Sweat-Based Glucose Monitoring Device With Multistage Transdermal Drug Delivery Module”	South Korea	Lee et al [55]
3 (90)	8 (223)	“Autonomous Sweat Extraction and Analysis Applied to Cystic Fibrosis and Glucose Monitoring Using a Fully Integrated Wearable Platform”	United States	Emaminejad et al [60]
4 (75)	14 (154)	“Soft, Smart Contact Lenses With Integrations of Wireless Circuits, Glucose Sensors, and Displays”	South Korea	Park et al [62]
5 (54)	27 (117)	“What Is the Economic Evidence for mHealth? A Systematic Review of Economic Evaluations of mHealth Solutions”	United States	Iribarren et al [63]
6 (53)	25 (121)	“Real-Time Continuous Glucose Monitoring in Adults With Type 1 Diabetes and Impaired Hypoglycaemia Awareness or Severe Hypoglycaemia Treated With Multiple Daily Insulin Injections (HypoDE): A Multicentre, Randomised Controlled Trial”	Germany and the United States	Heinemann et al [64]
6 (53)	72 (72)	“A Longitudinal Big Data Approach for Precision Health”	United States and Australia	Schüssler-Fiorenza Rose et al [65]
8 (49)	37 (110)	“Enzyme-Based Glucose Sensor: From Invasive to Wearable Device”	South Korea	Lee et al [66]
9 (47)	75 (70)	“Automated Diabetic Retinopathy Detection in Smartphone-Based Fundus Photography Using Artificial Intelligence”	India	Rajalakshmi et al [67]
10 (46)	77 (68)	“Cloud and IoT Based Disease Prediction and Diagnosis System for Healthcare Using Fuzzy Neural Classifier”	India	Kumar et al [68]

^a^TC_2021_: number of citations from the Web of Science Core Collection from its publication year to the end of 2021.

“A Graphene-Based Electrochemical Device With Thermoresponsive Microneedles for Diabetes Monitoring and Therapy” by Lee et al [54] was written by 12 authors from 5 institutions in South Korea and the United States, with a C_2021_ of 186 (ranked first) and a TC_2021_ of 705 (ranked first). In this study, Lee et al [54] enhanced the electrochemical activity of graphene combined with a gold mesh to create a wearable patch useful for determining sweat glucose levels as part of diabetes monitoring. In addition, this wearable can be thermally activated to release drugs according to a feedback strategy as the device can be connected to a wireless unit for data transmission.

“Wearable/Disposable Sweat-Based Glucose Monitoring Device With Multistage Transdermal Drug Delivery Module” by Lee et al [55] was written by 10 authors from 2 institutions in South Korea, with a C_2021_ of 143 (ranked second) and a TC_2021_ of 358 (ranked third). In this article, Lee et al [55] presented a wearable patch using sweat as a noninvasive method to measure glucose levels mainly based on heat, temperature, pH, and humidity sensors with a wireless data communication system for transdermal medication release, which is beneficial for the treatment of patients with diabetes.

“Autonomous Sweat Extraction and Analysis Applied to Cystic Fibrosis and Glucose Monitoring Using a Fully Integrated Wearable Platform” by Emaminejad et al [60] was written by 14 authors from 5 institutions in the United States, with a C_2021_ of 90 (ranked third) and a TC_2021_ of 223 (ranked eighth). In this study, Emaminejad et al [60] developed a wearable platform mainly integrated with sensors and a Bluetooth interface to connect the system to a mobile phone, inducing sweat secretion to provide a sufficient sweat sample to measure various characteristics, especially sweat glucose values. It is useful for the noninvasive continuous monitoring of patients with diabetes or prediabetes.

“Soft, Smart Contact Lenses With Integrations of Wireless Circuits, Glucose Sensors, and Displays” by Park et al [62] was written by 13 authors from 2 institutions in South Korea: Ulsan National Institute of Science and Technology and Sungkyunkwan University. This paper had a C_2021_ of 75 (ranked 4th) and a TC_2021_ of 154 (ranked 14th). The authors developed a smart contact lens to monitor tear glucose levels by integrating sensors, wireless communication components, and a display to visualize the measurement results, which is useful for patients with diabetes.

“What Is the Economic Evidence for mHealth? A Systematic Review of Economic Evaluations of mHealth Solutions” by Iribarren et al [63] was written by 4 authors from 3 institutions in the United States: University of Washington, Columbia University, and New York-Presbyterian Hospital. This paper had a C_2021_ of 54 (ranked 5th) and a TC_2021_ of 117 (ranked 27th). The authors emphasized the increasing economic evidence for mHealth as a cost-effective way to implement intervention activities, finding that the most common health conditions in the economic evaluations analyzed were outpatient visits, cardiovascular disease, and diabetes.

### Top 10 Most Frequent Author Keywords

The keywords used by the authors provide a reasonable description of the topics covered in the articles and indicate the directions in which the researchers are moving. The top 10 author keywords were determined as shown in Table 8.

**Table 8:** Top 10 most frequent author keywords (N=1277).

Author keywords	TP^a^, n (%)	Ranking	Hot spot
Telemedicine	122 (9.55)	1	Remote health care services
Self-management	98 (6.76)	2	Education and self-care activities
Smartphone	85 (6.66)	3	Innovative mobile app solutions
Mobile phone	66 (5.17)	4	Innovative mobile app solutions
Physical activity	52 (4.07)	5	Education and self-care activities
Mobile applications	41 (3.21)	6	Innovative mobile app solutions
Machine learning	37 (2.90)	7	Innovative mobile app solutions
Self-care	33 (2.58)	8	Education and self-care activities
Telehealth	33 (2.58)	8	Remote health care services
Mobile apps	31 (2.43)	10	Innovative mobile app solutions

^a^TP: total number of articles.

The author keywords were manually categorized with the support of the experts mentioned previously, and 3 groups were defined as the main hot spots of mHealth for diabetes: remote health care services, education and self-care activities, and innovative mobile app solutions. The keywords *telemedicine* and *telehealth* in positions 1 and 8, respectively, are the supporting keywords for the first hot spot, named “remote healthcare services.” The keywords *self-management*, *physical activity*, and *self-care*, which occupy positions 2, 5, and 8, respectively, are the supporting keywords for the second hot spot, “education and self-care activities.” Finally, the keywords *smartphone*, *mobile phone*, *mobile applications*, *machine learning*, and *mobile apps*, occupying positions 3, 4, 6, 7, and 10, respectively, are the supporting keywords for the third hot spot, “innovative mobile app solutions.”

## Discussion

### Principal Findings

The extensive deployment of mobile technologies has simplified access to health care services, especially for diabetes management. mHealth for diabetes is a potential facilitator to reduce costs and overcome geographical and temporal barriers to support people with diabetes in their disease control. Activities such as education, counseling, intervention, monitoring, medication administration, dietary intake, and physical activity can be supported by mHealth for diabetes.

Between 1998 and 2021, a total of 1574 articles on mHealth for diabetes were published in SCI Expanded, 1549 (98.41%) of which were written in English and 20 (1.27%) of which were written in German. The average number of citations for an article from the year of publication until 2021 was 15, and the maximum number of citations for an article was 705. It takes approximately 8 years for the CPP to reach a plateau. In total, 491 journals published articles related to mHealth for diabetes in 92 Web of Science categories. The most productive categories were Health Care Sciences & Services with 25.98% (409/1574) of the articles followed by Medical Informatics with 23% (362/1574) of the articles and Endocrinology & Metabolism with 16.65% (262/1574) of the articles. The most productive journals were *JMIR mHealth and uHealth* with 7.94% (125/1574) of the articles followed by the *Journal of Medical Internet Research* with 5.84% (92/1574) of the articles and *Diabetes Technology & Therapeutics* with 4.38% (69/1574) of the articles. The 1574 articles on mHealth for diabetes were published by authors from 90 countries, of which 1129 (71.73%) were single-country articles with authors from 62 countries, whereas 445 (28.27%) were international collaborative articles with authors from 83 countries.

The most productive countries in terms of publications were the United States with 35.96% (566/1574) of the articles followed by the United Kingdom with 10.93% (172/1574), China with 8.7% (137/1574), Australia with 6.73% (106/1574), South Korea with 6.04% (95/1574), Germany with 5.02% (79/1574), India with 4.64% (73/1574), Spain with 4.57% (72/1574), Canada with 4.45% (70/1574), and the Netherlands with 3.81% (60/1574). Since 2008, the United States has been in the lead with a significant upward trend. This becomes relevant when comparing these countries with respect to the diabetes-related health expenditure. It is estimated that these countries will maintain or slightly increase their diabetes-related health expenditure between 2021 and 2045 and, in some cases, such as South Korea, Germany, Spain, and the Netherlands, decrease it. This may indicate that the measures currently in place are having an impact on preventing new problems arising from the disease and new patients. In fact, it is expected that between 2030 and 2045, the number of adults aged between 20 and 79 years with diabetes in South Korea, Germany, and the Netherlands will decrease.

Regarding institutions, of the 1574 articles, 371 (23.57%) came from a single institution, whereas 1203 (76.43%) came from interinstitutional collaborations. The top 3 most productive institutions came from the United States, led by Harvard University with 3.88% (61/1574) of the articles followed by Stanford University with 2.16% (34/1574) and the University of Michigan with 1.65% (26/1574). The 1574 articles on mHealth for diabetes were written by 7922 authors. The APP was 6.5, whereas the maximum number of authors in an article was 73. The most productive authors were E Arsand with 18 articles followed by HS Kim with 15, X Li with 13, and E Renard and S Del Favero with 12. In terms of publication performance, which considered publication potential and publication characteristics, HS Kim had the greatest publication potential, followed by CC Quinn, CG Parkin, and JD Piette. A potential bias in the authorship analysis can be caused by different authors with the same name or the use of different names by the same author over time [69]. For example, in mHealth for diabetes research, author names such as Hee-Seung Kim, Hun-Sung Kim, Hee-Sung Kim, Hee-Seon Kim, Hyun Seung Kim, and Hwa Sun Kim could be considered as HS Kim. Except for HS Kim, CC Quinn with the *Y*-index (14, π/4) had both the highest publication potential with a *j* of 14 and the highest CPP_2021_ for first-author articles and corresponding-author articles, as previously shown in Table 6.

On the other hand, the most cited articles were the 2016 article by Lee et al [54] titled “A Graphene-Based Electrochemical Device With Thermoresponsive Microneedles for Diabetes Monitoring and Therapy” with a TC_2021_ of 705, followed by the 2009 article by Krishna et al [46] titled “Healthcare via Cell Phones: A Systematic Review” with a TC_2021_ of 550 and the 2017 article by Lee et al [55] titled “Wearable/Disposable Sweat-Based Glucose Monitoring Device With Multistage Transdermal Drug Delivery Module” with a TC_2021_ of 358. The top 10 articles with the highest impact were published by 96 authors from 30 institutions from the United States, South Korea, India, Australia, and Germany. The top 3 articles with the highest impact were “A Graphene-Based Electrochemical Device With Thermoresponsive Microneedles for Diabetes Monitoring and Therapy” by Lee et al [54] followed by “Wearable/Disposable Sweat-Based Glucose Monitoring Device With Multistage Transdermal Drug Delivery Module” by Lee et al [55] and “Autonomous Sweat Extraction and Analysis Applied to Cystic Fibrosis and Glucose Monitoring Using a Fully Integrated Wearable Platform” by Emaminejad et al [60].

### Research Hot Spots

The current research hot spots in mHealth for diabetes were determined by analyzing the top 10 most frequent author keywords (Table 8), and 3 hot spots were manually identified with help from experts in the field: remote health care services, education and self-care activities, and innovative mobile app solutions.

Remote health care services describe the delivery of health care services using a variety of mobile technologies, such as mobile apps, smartphones, and videoconferencing. These services include medical consultations, diagnosis, monitoring, and more, giving patients the opportunity to interact with a variety of medical specialists without the need to travel or long waiting times. Telemedicine and telehealth play an important role in the delivery of remote health care services. While telemedicine specifically refers to clinical services delivered remotely, telehealth encompasses a broader range of health care activities, including clinical care, patient education, health promotion, and administrative functions. Telehealth comprises both clinical and nonclinical services. Solutions to improve remote services are rapidly evolving with the support of digital health technologies such as smart devices, digital platforms, and sensors that can work together in a digital framework to simultaneously operate with multiple diabetes control and management activities, such as consultation, monitoring, and interventions. For example, Lian et al [70] demonstrated the effectiveness of a telehealth framework to support diabetes care during the COVID-19 pandemic, becoming an alternative to traditional consultations. In addition, Varnfield et al [71] showed the value of an mHealth platform for women with gestational diabetes mellitus by providing tools for multidisciplinary care coordination, including an app, a clinician portal, and cloud data storage, whereas Spoladore et al [72] presented an ontology-based telehealth platform to promote healthy food intake and physical activity in older adults with chronic diseases, providing appropriate suggestions based on health conditions. Similarly, May et al [73] highlighted the opportunities that digital health technologies offer to clinical practice, improving care delivery and patient engagement.

On the other hand, the education and self-care activities hot spot describes self-management actions that empower patients to take an active role in managing their health, promoting wellness and optimal health outcomes. These actions include a range of behaviors and strategies designed to promote health, prevent diabetes, and effectively manage existing health problems. Educational activities to help patients learn about their health conditions are critical in achieving a healthy lifestyle, promoting adequate physical activity and nutrition as well as treatment options and medications. In addition, it is important to mention that health education empowers patients with the knowledge and skills to make informed decisions about their health and participate effectively in their own care. In this sense, the use of digital technologies, including information and communications technologies, mobile technologies, smart devices, and SMS text messaging, is promoted. For example, Clark et al [74] demonstrated the benefits of using mHealth for diabetes self-management, including education, motivation, and intervention activities for individuals with diabetes distress. The impact of mobile technology interventions has also been evaluated by Chin-Jung et al [75], who found improvements in glucose levels and quality of life. Meanwhile, Whelan et al [76] explored people’s engagement in a digital lifestyle in individuals at high risk of developing type 2 diabetes, finding that both real-time behavioral and physiological feedback have a positive impact on health. In addition, Rodriguez et al [77] developed a customized and automated SMS text messaging strategy based on computational tools to involve individuals in a digital diabetes prevention program. Similarly, McGill et al [78] created an SMS text messaging intervention strategy for adolescents with type 1 diabetes, resulting in improved glycemic control. In addition, Tian et al [79] compared the effectiveness of glycemic control in women with gestational diabetes using social media through a group chat app. They demonstrated the value of instant messaging to improve glycemic control through educational and lifestyle intervention programs. To successfully integrate mHealth into diabetes care activities, changes in health care education and clinical practice are needed [80], and collaboration between patients and health care professionals is fundamental.

Finally, the innovative mobile app solutions hot spot describes the capabilities of mobile technologies such as smartphones and wearables managed by apps to improve diabetes management. These apps integrate advances in artificial intelligence, machine learning, and virtual reality to empower patients to effectively manage their health and help health care professionals improve their services. Furthermore, studies on the economic impact of mobile apps were also present in this hot spot. For example, Ramchandani [81] identified the benefits of internet-based guidance and diabetes-related apps to provide personalized support in real time, reducing the need for health care professionals to assist patients at any time. In addition, Tsuji et al [82] determined a favorable impact on the cost-effectiveness of a mobile app for continuous glucose monitoring using a Markov model to develop a cost simulation, whereas Fu et al [83] evaluated 4 top-rated diabetes apps for patients with type 2 diabetes and found that the future design of diabetes apps should consider patient motivation and a key heuristic design approach with supporting functions. Similarly, Khurana et al [84] validated the reliability of a mobile app to remotely monitor visual acuity and metamorphopsia. In a separate study, Shah et al [85] showed the utility of a direct ophthalmoscope-based smartphone camera for screening diabetic retinopathy. In the same way, Ludwig et al [86] managed to obtain low-resolution fundus images using a mobile phone and an indirect ophthalmoscope lens adapter, which were processed by a deep learning algorithm to detect diabetic retinopathy. On the other hand, diabetic foot ulcers have also been studied. In this context, Oe et al [87] proposed a mobile thermograph attached to a smartphone to prevent diabetic foot ulcers. Furthermore, Goyal et al [88] revealed the efficacy of a robust deep learning model to detect and locate diabetic foot ulcers in real time using a smartphone app, and Lin et al [89] validated the contribution of using wireless wearable near-infrared spectroscopy to predict wound prognosis in diabetic foot ulcers.

Other studies included advances in machine learning to propose solutions for diabetes control. For example, Kumar et al [90] demonstrated the effectiveness of a machine learning model combined with a mobile app to diagnose diseases such as diabetes, heart disease, and COVID-19, facilitating physician disease identification for appropriate treatment. Moreover, Bahadur et al [91] used human activity recognition based on machine learning algorithms to propose an efficient model to recognize and monitor 13 activities associated with diabetes symptoms to determine whether a person has diabetes mellitus. In another study, McVean and Miller [92] reported positive results from an insulin pump system with smartphone connectivity for the treatment of type 1 diabetes.

### Conclusions

This study presents the dynamics of scientific publications in mHealth for diabetes through a scientometric analysis based on a CTI approach. CTI added value with a methodology supported by experts who provided feedback during the research process and helped consolidate different elements, including the search queries. The SCI Expanded database was used to collect data and provide insights into the evolution of the field according to the following: publication language, publication output characteristics, Web of Science categories and journals, country and institution publication performance, author publication performance, citation history of the most cited articles, and the articles with the highest impact according to their citation behavior.

This approach provides a comprehensive knowledge panorama of research productivity in mHealth for diabetes, identifying new insights and opportunities for R&D and innovation, including collaboration with other entities, new areas of specialization, and human resource development. It enables the identification of the productivity and impact of the field, comprising leading authors, countries, and journals; focus areas; and highly influential papers. In the top 10 most productive countries in terms of publications related to mHealth for diabetes, the prevalence of adult patients with diabetes will increase slightly by 2045. These countries exhibit strong efforts to discourage diabetes incidence. Researchers can develop collaborations and strategies by identifying key players in countries that contribute significantly to the field of mHealth for diabetes, creating opportunities for better diabetes management.

From a global perspective, the use of digital technologies to improve health conditions and services aims to reduce health disparities. Digital health is evolving rapidly; insights obtained indicate important efforts on health informatics, telemedicine, telecare, and eHealth to improve diabetes care and its prevention. Notable progress has been identified in the opportunities that digital health technologies offer to implement multiple activities to support diabetes management.

In the future, mHealth for diabetes will place the patient at the center of the ecosystem. Apps will provide user-friendly experiences and personalized recommendations based on the patients’ specific circumstances and context. The use of machine learning will help prevent the development of diabetes, detect patterns that lead to the disease’s development, and optimize treatment plans. The implementation of decision support systems in mobile apps to assist both patients and health care professionals in making informed decisions about diabetes management is a notable development for the future. Physical activity tracking and food plans will be supported using sensors, smartphones, wearables, and machine learning to provide personalized insights and recommendations based on personal health characteristics. Tailored medications for diabetes management are expected to be available through 3D printing technology. Various mobile technologies, electronic health records, the Internet of Things, and other health care infrastructure will be integrated with a focus on creating interoperable systems that will enable continuous patient monitoring for efficient diabetes management, facilitating timely action in real time. It is expected that future analyses of large data sets generated by mHealth technologies will lead to the inclusion of mental health in the treatment of diabetes. In addition, mobile technology is expected to benefit the patients’ caregivers and relatives.

Understanding the research dynamics in mHealth for diabetes, such as the evolution of the field and its current state, as well as providing future insights, enables researchers, health care professionals, and decision makers to focus their resources and efforts on meaningful contributions in which a promising future for mHealth for diabetes science is envisioned. Scholars are encouraged to apply this approach to study the scientific and technological dynamics of this and other fields of study, facilitating a continuous monitoring of the scientific and technological environment, a crucial activity for a strategic vision.

## Appendix

Multimedia Appendix 1 Search query.

## Abbreviations

APP: average number of authors per article
CPC: number of international collaborative articles
CPI: number of interinstitutional collaborative articles
CPP: average number of citations per publication
CTI: competitive technology intelligence
FP: number of first-author articles
IF: impact factor
IPC: number of single-country articles
IPI: number of single-institution articles
JCR: Journal Citation Reports
mHealth: mobile health
R&D: research and development
RP: number of corresponding-author articles
SCI: Science Citation Index
SP: number of single-author articles
TC: total number of citations from the year of publication to the end of a specific year
TP: total number of articles

## References

[1] Number of smartphone mobile network subscriptions worldwide from 2016 to 2023, with forecasts from 2023 to 2028. Statista.

[2] Number of apps available in leading app stores as of 3rd quarter 2022. Statista.

[3] Number of mobile app downloads worldwide from 2016 to 2023. Statista.

[4] Number of apps released worldwide between 3rd quarter 2019 and 2nd quarter 2020, by category. Statista.

[5] Most popular app categories worldwide during 3rd quarter 2020, by reach. Statista.

[6] Luxton DD, McCann RA, Bush NE, Mishkind MC, Reger GM (2011). mHealth for mental health: integrating smartphone technology in behavioral healthcare. Prof Psychol Res Pract.

[7] Hamine S, Gerth-Guyette E, Faulx D, Green BB, Ginsburg AS (2015). Impact of mHealth chronic disease management on treatment adherence and patient outcomes: a systematic review. J Med Internet Res.

[8] Silva BM, Rodrigues JJ, de la Torre Díez I, López-Coronado M, Saleem K (2015). Mobile-health: a review of current state in 2015. J Biomed Inform.

[9] IDF Diabetes Atlas 2021. International Diabetes Federation.

[10] Salvador MR, Lopez-Martinez RE (2000). Cognitive structure of research: scientometric mapping in sintered materials. Res Eval.

[11] Yin CY (2015). Measuring organizational impacts by integrating competitive intelligence into executive information system. J Intell Manuf.

[12] Fourati-Jamoussi F, Narcisse Niamba C (2016). An evaluation of business intelligence tools: a cluster analysis of users’ perceptions. J Intell Stud Bus.

[13] Semerkova LN, Zaretskiy AP, Divnenko ZA, Grosheva ES, Vishnevskaya GV (2017). Application of information technologies in competitive intelligence. Proceedings of the XX IEEE International Conference on Soft Computing and Measurements.

[14] Du Toit AS (2015). Competitive intelligence research: an investigation of trends in the literature. J Intell Stud Bus.

[15] Ali Köseoglu M, Ross G, Okumus F (2016). Competitive intelligence practices in hotels. Int J Hosp Manag.

[16] Saba M, Bou Saba P, Harfouche A (2018). Hidden facets of IT projects are revealed only after deployment: the case of French agricultural cooperatives. Inf Technol People.

[17] Rothberg HN, Erickson GS (2017). Big data systems: knowledge transfer or intelligence insights?. J Knowl Manag.

[18] Rodriguez-Salvador M, Castillo-Valdez PF (2021). Integrating science and technology metrics into a competitive technology intelligence methodology. J Intell Stud Bus.

[19] Rodríguez-Salvador M, Rio-Belver RM, Garechana-Anacabe G (2017). Scientometric and patentometric analyses to determine the knowledge landscape in innovative technologies: the case of 3D bioprinting. PLoS One.

[20] Safa M, Shahi A, Haas CT, Fiander-McCann D, Safa M, Hipel K, MacGillivray S (2015). Competitive intelligence (CI) for evaluation of construction contractors. Autom Constr.

[21] Fernández-Arias MP, Quevedo-Cano P, Hidalgo-Nuchera A (2016). Uso de la inteligencia competitiva en los procesos de colaboración en el sector farmacéutico español. El Profesional De La Información.

[22] Garcia-Alsina M, Cobarsí-Morales J, Ortoll E (2015). Competitive intelligence theoretical framework and practices: the case of Spanish universities. Aslib J Inf Manag.

[23] Peters I, Frodeman R, Wilsdon J, European Commission, Directorate-General for Research and Innovation (2017). Next-generation metrics: responsible metrics and evaluation for open science. Publications Office of the European Union.

[24] Aina TA, Cooke L, Stephens D (2016). Methodology for evaluating CI software packages. Bus Inf Rev.

[25] Pargaonkar YR (2016). Leveraging patent landscape analysis and IP competitive intelligence for competitive advantage. World Pat Inf.

[26] Ho YS, Shekofteh M (2021). Performance of highly cited multiple sclerosis publications in the Science Citation Index expanded: a scientometric analysis. Mult Scler Relat Disord.

[27] Science citation index-expanded. Clarivate.

[28] Garfield E (1990). Keywords PlusTM: ISI’s breakthrough retrieval method. Part 1. Expanding your searching power on current contents on diskette. Curr Content.

[29] Fu HZ, Ho YS (2015). Top cited articles in thermodynamic research. J Eng Thermophys.

[30] Wang MH, Ho YS (2011). Research articles and publication trends in environmental sciences from 1998 to 2009. Arch Environ Sci.

[31] Fu HZ, Wang MH, Ho YS (2012). The most frequently cited adsorption research articles in the Science Citation Index (Expanded). J Colloid Interface Sci.

[32] Li Z, Ho YS (2008). Use of citation per publication as an indicator to evaluate contingent valuation research. Scientometrics.

[33] Chiu WT, Ho YS (2005). Bibliometric analysis of homeopathy research during the period of 1991 to 2003. Scientometrics.

[34] Ho YS (2012). Top-cited articles in chemical engineering in science citation index expanded: a bibliometric analysis. Chin J Chem Eng.

[35] Wang MH, Fu HZ, Ho YS (2011). Comparison of universities' scientific performance using bibliometric indicators. Malays J Libr Inf Sci.

[36] Ho YS (2012). The top-cited research works in the Science Citation Index Expanded. Scientometrics.

[37] Hsu YH, Ho YS (2014). Highly cited articles in health care sciences and services field in Science Citation Index Expanded. A bibliometric analysis for 1958 - 2012. Methods Inf Med.

[38] Ho YS, Mukul SA (2021). Publication performance and trends in mangrove forests: a bibliometric analysis. Sustainability.

[39] Ho YS (2014). A bibliometric analysis of highly cited articles in materials science. Curr Sci.

[40] Ho YS, Siu E, Chuang KY (2016). A bibliometric analysis of dengue-related publications in the Science Citation Index Expanded. Future Virol.

[41] Pouris A, Ho YS (2017). A bibliometric analysis of research on Ebola in Science Citation Index Expanded. S Afr J Sci.

[42] Li Y, Wang X, Thomsen JB, Nahabedian MY, Ishii N, Rozen WM, Long X, Ho YS (2020). Research trends and performances of breast reconstruction: a bibliometric analysis. Ann Transl Med.

[43] Giannoudis PV, Chloros GD, Ho YS (2021). A historical review and bibliometric analysis of research on fracture nonunion in the last three decades. Int Orthop.

[44] Chong Y, Long X, Ho YS (2021). Scientific landscape and trend analysis of keloid research: a 30-year bibliometric review. Ann Transl Med.

[45] Farooq M, Khan AU, El-Adawy H, Mertens-Scholz K, Khan I, Neubauer H, Ho YS (2022). Research trends and hotspots of Q fever research: a bibliometric analysis 1990-2019. Biomed Res Int.

[46] Krishna S, Boren SA, Balas EA (2009). Healthcare via cell phones: a systematic review. Telemed J E Health.

[47] Quinn CC, Clough SS, Minor JM, Lender D, Okafor MC, Gruber-Baldini A (2008). WellDoc mobile diabetes management randomized controlled trial: change in clinical and behavioral outcomes and patient and physician satisfaction. Diabetes Technol Ther.

[48] Ho YS (2021). A bibliometric analysis of highly cited publications in Web of Science category of emergency medicine. Signa Vitae.

[49] Riesenberg D, Lundberg GD (1990). The order of authorship: who's on first?. JAMA.

[50] Total diabetes-related health expenditure, USD million. International Diabetes Federation Diabetes Atlas 10th Edition 2021.

[51] (2021). Diabetes estimates (20-79 y): people with diabetes, in 1,000s. International Diabetes Federation Diabetes Atlas 10th Edition 2021.

[52] (2021). Diabetes estimates (20-79 y): age-adjusted comparative prevalence of diabetes, %. International Diabetes Federation Diabetes Atlas 10th Edition 2021.

[53] Ho YS, Hartley J (2016). Classic articles published by American scientists (1900-2014): a bibliometric analysis. Curr Sci.

[54] Lee H, Choi TK, Lee YB, Cho HR, Ghaffari R, Wang L, Choi HJ, Chung TD, Lu N, Hyeon T, Choi SH, Kim DH (2016). A graphene-based electrochemical device with thermoresponsive microneedles for diabetes monitoring and therapy. Nat Nanotechnol.

[55] Lee H, Song C, Hong YS, Kim M, Cho HR, Kang T, Shin K, Choi SH, Hyeon T, Kim DH (2017). Wearable/disposable sweat-based glucose monitoring device with multistage transdermal drug delivery module. Sci Adv.

[56] Russell SJ, El-Khatib FH, Sinha M, Magyar KL, McKeon K, Goergen LG, Balliro C, Hillard MA, Nathan DM, Damiano ER (2014). Outpatient glycemic control with a bionic pancreas in type 1 diabetes. N Engl J Med.

[57] Cafazzo JA, Casselman M, Hamming N, Katzman DK, Palmert MR (2012). Design of an mHealth app for the self-management of adolescent type 1 diabetes: a pilot study. J Med Internet Res.

[58] Quinn CC, Shardell MD, Terrin ML, Barr EA, Ballew SH, Gruber-Baldini AL (2011). Cluster-randomized trial of a mobile phone personalized behavioral intervention for blood glucose control. Diabetes Care.

[59] Liang X, Wang Q, Yang X, Cao J, Chen J, Mo X, Huang J, Wang L, Gu D (2011). Effect of mobile phone intervention for diabetes on glycaemic control: a meta-analysis. Diabet Med.

[60] Emaminejad S, Gao W, Wu E, Davies ZA, Yin Yin Nyein H, Challa S, Ryan SP, Fahad HM, Chen K, Shahpar Z, Talebi S, Milla C, Javey A, Davis RW (2017). Autonomous sweat extraction and analysis applied to cystic fibrosis and glucose monitoring using a fully integrated wearable platform. Proc Natl Acad Sci U S A.

[61] Pagoto S, Schneider K, Jojic M, DeBiasse M, Mann D (2013). Evidence-based strategies in weight-loss mobile apps. Am J Prev Med.

[62] Park J, Kim J, Kim SY, Cheong WH, Jang J, Park YG, Na K, Kim YT, Heo JH, Lee CY, Lee JH, Bien F, Park JU (2018). Soft, smart contact lenses with integrations of wireless circuits, glucose sensors, and displays. Sci Adv.

[63] Iribarren SJ, Cato K, Falzon L, Stone PW (2017). What is the economic evidence for mHealth? A systematic review of economic evaluations of mHealth solutions. PLoS One.

[64] Heinemann L, Freckmann G, Ehrmann D, Faber-Heinemann G, Guerra S, Waldenmaier D, Hermanns N (2018). Real-time continuous glucose monitoring in adults with type 1 diabetes and impaired hypoglycaemia awareness or severe hypoglycaemia treated with multiple daily insulin injections (HypoDE): a multicentre, randomised controlled trial. Lancet.

[65] Schüssler-Fiorenza Rose SM, Contrepois K, Moneghetti K, Zhou W, Mishra T, Mataraso S, Dagan-Rosenfeld O, Ganz A, Dunn J, Hornburg D, Rego S, Perelman D, Ahadi S, Sailani M, Zhou Y, Leopold S, Chen J, Ashland M, Christle J, Avina M, Limcaoco P, Ruiz C, Tan M, Butte AJ, Weinstock GM, Slavich GM, Sodergren E, McLaughlin TL, Haddad F, Snyder MP (2019). A longitudinal big data approach for precision health. Nat Med.

[66] Lee H, Hong YJ, Baik S, Hyeon T, Kim DH (2018). Enzyme-based glucose sensor: from invasive to wearable device. Adv Healthc Mater.

[67] Rajalakshmi R, Subashini R, Anjana RM, Mohan V (2018). Automated diabetic retinopathy detection in smartphone-based fundus photography using artificial intelligence. Eye (Lond).

[68] Kumar PM, Lokesh S, Varatharajan R, Chandra Babu G, Parthasarathy P (2018). Cloud and IoT based disease prediction and diagnosis system for healthcare using Fuzzy neural classifier. Future Gener Comput Syst.

[69] Chiu WT, Ho YS (2007). Bibliometric analysis of tsunami research. Scientometrics.

[70] Lian X, Dalan R, Seow CJ, Liew H, Jong M, Chew D, Lim B, Lin A, Goh E, Goh C, Othman NB, Tan L, Boehm BO (2021). Diabetes care during COVID-19 pandemic in Singapore using a telehealth strategy. Horm Metab Res.

[71] Varnfield M, Redd C, Stoney RM, Higgins L, Scolari N, Warwick R, Iedema J, Rundle J, Dutton W (2021). M♡THer, an mHealth system to support women with gestational diabetes mellitus: feasibility and acceptability study. Diabetes Technol Ther.

[72] Spoladore D, Colombo V, Arlati S, Mahroo A, Trombetta A, Sacco M (2021). An ontology-based framework for a telehealthcare system to foster healthy nutrition and active lifestyle in older adults. Electronics.

[73] May SG, Huber C, Roach M, Shafrin J, Aubry W, Lakdawalla D, Kane JM, Forma F (2021). Adoption of digital health technologies in the practice of behavioral health: qualitative case study of glucose monitoring technology. J Med Internet Res.

[74] Clark TL, Gallo L, Euyoque JA, Philis-Tsimikas A, Fortmann A (2020). Does diabetes distress influence clinical response to an mHealth diabetes self-management education and support intervention?. Diabetes Educ.

[75] Chin-Jung L, Hsiao-Yean C, Yeu-Hui C, Kuan-Chia L, Hui-Chuan H (2021). Effects of mobile health interventions on improving glycemic stability and quality of life in patients with type 1 diabetes: a meta-analysis. Res Nurs Health.

[76] Whelan ME, Denton F, Bourne CL, Kingsnorth AP, Sherar LB, Orme MW, Esliger DW (2021). A digital lifestyle behaviour change intervention for the prevention of type 2 diabetes: a qualitative study exploring intuitive engagement with real-time glucose and physical activity feedback. BMC Public Health.

[77] Rodriguez DV, Lawrence K, Luu S, Yu JL, Feldthouse DM, Gonzalez J, Mann D (2021). Development of a computer-aided text message platform for user engagement with a digital Diabetes Prevention Program: a case study. J Am Med Inform Assoc.

[78] McGill DE, Laffel LM, Volkening LK, Butler DA, Levy WL, Wasserman RM, Anderson BJ (2020). Text message intervention for teens with type 1 diabetes preserves HbA1c: results of a randomized controlled trial. Diabetes Technol Ther.

[79] Tian Y, Zhang S, Huang F, Ma L (2021). Comparing the efficacies of telemedicine and standard prenatal care on blood glucose control in women with gestational diabetes mellitus: randomized controlled trial. JMIR Mhealth Uhealth.

[80] Bradway M, Morris RL, Giordanengo A, Årsand E (2020). How mHealth can facilitate collaboration in diabetes care: qualitative analysis of co-design workshops. BMC Health Serv Res.

[81] Ramchandani N (2019). Virtual coaching to enhance diabetes care. Diabetes Technol Ther.

[82] Tsuji S, Ishikawa T, Morii Y, Zhang H, Suzuki T, Tanikawa T, Nakaya J, Ogasawara K (2020). Cost-effectiveness of a continuous glucose monitoring mobile app for patients with type 2 diabetes mellitus: analysis simulation. J Med Internet Res.

[83] Fu HN, Rizvi RF, Wyman JF, Adam TJ (2020). Usability evaluation of four top-rated commercially available diabetes apps for adults with type 2 diabetes. Comput Inform Nurs.

[84] Khurana RN, Hoang C, Khanani AM, Steklov N, Singerman LJ (2021). A smart mobile application to monitor visual function in diabetic retinopathy and age-related macular degeneration: the CLEAR study. Am J Ophthalmol.

[85] Shah D, Dewan L, Singh A, Jain D, Damani T, Pandit R, Porwal AC, Bhatnagar S, Shrishrimal M, Patel A (2021). Utility of a smartphone assisted direct ophthalmoscope camera for a general practitioner in screening of diabetic retinopathy at a primary health care center. Indian J Ophthalmol.

[86] Ludwig CA, Perera C, Myung D, Greven MA, Smith SJ, Chang RT, Leng T (2020). Automatic identification of referral-warranted diabetic retinopathy using deep learning on mobile phone images. Transl Vis Sci Technol.

[87] Oe M, Tsuruoka K, Ohashi Y, Takehara K, Noguchi H, Mori T, Yamauchi T, Sanada H (2021). Prevention of diabetic foot ulcers using a smartphone and mobile thermography: a case study. J Wound Care.

[88] Goyal M, Reeves ND, Rajbhandari S, Yap MH (2019). Robust methods for real-time diabetic foot ulcer detection and localization on mobile devices. IEEE J Biomed Health Inform.

[89] Lin BS, Chang CC, Tseng YH, Li JR, Peng YS, Huang YK (2020). Using wireless near-infrared spectroscopy to predict wound prognosis in diabetic foot ulcers. Adv Skin Wound Care.

[90] Kumar N, Narayan Das N, Gupta D, Gupta K, Bindra J (2021). Efficient automated disease diagnosis using machine learning models. J Healthc Eng.

[91] Bahadur EH, Masum AK, Barua A, Uddin MZ (2021). Active sense: early staging of Non-Insulin Dependent Diabetes Mellitus (NIDDM) hinges upon recognizing daily activity pattern. Electronics.

[92] McVean J, Miller J (2021). MiniMed780G Insulin pump system with smartphone connectivity for the treatment of type 1 diabetes: overview of its safety and efficacy. Expert Rev Med Devices.

