# Supplementary material for: Scientific Production Dynamics in mHealth for Diabetes: Scientometric Analysis
Source: JMIR Diabetes. 2024 Aug 22;9:e52196. doi: 10.2196/52196 (PMC11377915; doi:10.2196/52196)
Supplement: Multimedia Appendix 1 [file diabetes_v9i1e52196_app1.docx]

**Search Query Supplementary File**

Guidance for conducting this query is documented in the methodology section of this paper.

((TS=((“diabetes” or “diabetic” or “diabetology” or “glucose level” or “glucose levels” or “blood glucose” or “blood sugar” or “glycated hemoglobin” or “glycemic control” or “hemoglobin a1c” or “A1C” or “hemoglobinalc” or “hba1c” or “HgbA1c” or “hyperglycemia” or “hyperglycemics” or “hyperglycemic” or “hypoglycemia” or “hypoglycemias” or “hypoglycemic” or “hypoglycemics”) and (“app” or “apps” or “cellphone” or “cell phones” or “cell phone” or “cellular phone” or “cellular phones” or “cellphones” or “mhealth” or “m-health” or “mobile applications” or “mobile application” or “mobile device” or “mobile devices” or “mobile monitoring” or “mobile health” or “mobile healthcare” or “mobile intervention” or “mobile interventions” or “mobile phone” or “mobile phones” or “mobile systems” or “mobile system” or “mobile wireless” or “smart device” or “smart devices” or “smart phone” or “smart phones” or “smartphone” or “smartphones” or ((“wearable” or “wearables”) and (“electronic” or “electronics” or “technology” or “technologies” or “digital” or “smart” or “intelligent” or “intelligence” or “mobile” or “wireless” or “sensor” or “sensors” or “monitor” or “monitoring” or “tracker” or “trackers” or “self-management” or “self-care” or “self-control” or “portable”)) or “tablet technology” or ((“tablet” or “tablets”) and (“electronic” or “electronics” or “electronically” or “technological” or “technology” or “technologies” or “technical” or “digital” or “automatically” or “automatic” or “device” or “devices” or “smart” or “intelligent” or “intelligence” or “mobile” or “wireless”))))) NOT (TS=(“acute-phase” or “amyloid” or “adzuki” or “glycosidase” or “pollock” or “polyphenol” or “precursor” or “secretase” or “antiplasmin” or “mu(app)” or “peak pressure” or “polyphosphate” or “pK(a,app)” or “phosphatase” or “actinobacillus” or “APP/AuNPs” or “P-app” or “k(2,app)” or “k(app)” or “propranolol” or “APP-10” or “pyelonephritis” or “polysaccharide” or “K-m(app)” or “apolipoprotein” or “K(m)” or “polysaccharide” or “polypeptide” or “artemisia” or “agouti” or “K-I app” or “Delta g” or “mutations” or “zucker” or “APP/PS1” or “serum selenium” or “pulse pressure (APP)” or “patency” or “zygomycosis” or “BACE1” or “S-nitrosylation” or “phosphorylation” or “advanced practice providers” or “Ag/AgCl” or “Pi3K” or “DEGs” or “vanadium” or “pyrrole” or “oxidant” or “inhibitor” or “DPP4is” or “matrix tablet” or “tablet-dispensing” or “disintegrating tablet” or “capsule” or “ginkgo” or “alogliptin” or “benzoate” or “repaglinide” or “dissolution” or “pump tablet” or “pump tablets” or “tablet dosage” or “placebo” or “poly-DL-lactic” or “acetylation” or “acetyl” or “radical” or “hydrochloride” or “uniformity of tablets” or “paliperidone” or “monolayer” or “mitochondrial” or “number of tablets” or “single-tablet” or “orodispersible tablets” or “release tablet” or “formulation” or “oral medicines” or “metanol” or “sitagliptin” or “chlorine” or “ethambutol” or “denervation” or “vaspin” or “SYKFT” or “mimetic” or “dissolvable” or “HPLC” or “JQJTT” or “implantable devices” or “nanostructure” or “raman spectroscopy” or “TEOS” or “ACF” or “MoS2” or “Zn+2” or “OCMC” or “SPEs” or “CVD-grown” or “Dirac” or “UHPLC” or “polyimide” or “TENGs” or “PEDOT” or “WSNF” or “SCE” or “EBFCs” or “FPCB” or “NSCLC” or “HED” or “hydroxychloroquine” or “subfertile” or “tuber” or “cleansing” or “meses” or “PCOS” or “PICC” or “DKD” or “1AF” or “CIPN” or “POAF” or “hs-CRP” or “nCoV-19” or “CPB” or “MCCOD” or “PSG” or “L-Asp” or “MeSHDD” or “JoVE” or “D-3” or “TRAP” or “RASGD” or “mobile clinics” or “mobile clinic” or “JIF” or “mobile health promotion” or “mobile health unit” or “mobile healthcare service” or “mobile health service” or “health data linkage unit” or “mobile health camp” or “mobile health van”))) and PY=1998-2021 and DT=article
